# ADP-dependent glucokinase controls metabolic fitness in prostate cancer progression

**DOI:** 10.1186/s40779-023-00500-9

**Published:** 2023-12-12

**Authors:** Hang Xu, Yi-Fan Li, Xian-Yan-Ling Yi, Xiao-Nan Zheng, Yang Yang, Yan Wang, Da-Zhou Liao, Jia-Peng Zhang, Ping Tan, Xing-Yu Xiong, Xi Jin, Li-Na Gong, Shi Qiu, De-Hong Cao, Hong Li, Qiang Wei, Lu Yang, Jian-Zhong Ai

**Affiliations:** 1 Department of Urology, West China Hospital, Sichuan University, Chengdu, 610041 China; 2 Institute of Urology, West China Hospital, Sichuan University, Chengdu, 610041 China; 3 Animal Experimental Center, West China Hospital, Sichuan University, Chengdu, 610041 China; 4 Research Core Facility, West China Hospital, Sichuan University, Chengdu, 610041 China

**Keywords:** Prostate cancer (PCa), ADP-dependent glucokinase (ADPGK), Aldolase C (ALDOC), AMPK, Glycolysis

## Abstract

**Background:**

Cell metabolism plays a pivotal role in tumor progression, and targeting cancer metabolism might effectively kill cancer cells. We aimed to investigate the role of hexokinases in prostate cancer (PCa) and identify a crucial target for PCa treatment.

**Methods:**

The Cancer Genome Atlas (TCGA) database, online tools and clinical samples were used to assess the expression and prognostic role of ADP-dependent glucokinase (ADPGK) in PCa. The effect of ADPGK expression on PCa cell malignant phenotypes was validated in vitro and in vivo. Quantitative proteomics, metabolomics, and extracellular acidification rate (ECAR) and oxygen consumption rate (OCR) tests were performed to evaluate the impact of ADPGK on PCa metabolism. The underlying mechanisms were explored through ADPGK overexpression and knockdown, co-immunoprecipitation (Co-IP), ECAR analysis and cell counting kit-8 (CCK-8) assays.

**Results:**

ADPGK was the only glucokinase that was both upregulated and predicted worse overall survival (OS) in prostate adenocarcinoma (PRAD). Clinical sample analysis demonstrated that ADPGK was markedly upregulated in PCa tissues vs. non-PCa tissues. High ADPGK expression indicates worse survival outcomes, and ADPGK serves as an independent factor of biochemical recurrence. In vitro and in vivo experiments showed that ADPGK overexpression promoted PCa cell proliferation and migration, and ADPGK inhibition suppressed malignant phenotypes. Metabolomics, proteomics, and ECAR and OCR tests revealed that ADPGK significantly accelerated glycolysis in PCa. Mechanistically, ADPGK binds aldolase C (ALDOC) to promote glycolysis via AMP-activated protein kinase (AMPK) phosphorylation. ALDOC was positively correlated with ADPGK, and high ALDOC expression was associated with worse survival outcomes in PCa.

**Conclusions:**

In summary, ADPGK is a driving factor in PCa progression, and its high expression contributes to a poor prognosis in PCa patients. ADPGK accelerates PCa glycolysis and progression by activating ALDOC-AMPK signaling, suggesting that ADPGK might be an effective target and marker for PCa treatment and prognosis evaluation.

**Supplementary Information:**

The online version contains supplementary material available at 10.1186/s40779-023-00500-9.

## Background

Prostate cancer (PCa) is one of the most common malignancies in men [1, 2]. Radical prostatectomy (laparoscopic or robot-assisted approaches) and radiotherapy have remained the gold standard for localized PCa treatment [3]. In China, approximately half of PCa patients are diagnosed at the late stage. Under these conditions, radical resection cannot be performed. Androgen deprivation therapy (ADT) is the cornerstone of PCa treatments and is also the dominant therapy for advanced PCa [4]. However, with the continuous progression of the disease, most PCa patients who have received ADT will inevitably show progression to castration-resistant prostate cancer (CRPC) after 18–24 months [5]. CRPC is characterized by a poor prognosis and limited therapeutic efficacy [6]. Therefore, it is critical to address the limitations of clinical treatments for patients with advanced PCa by exploring the underlying molecular mechanism of PCa progression.

It was recently discovered that glucose metabolism in tumor cells is closely related to tumor growth, metastasis and drug resistance, and strategies to disturb tumor glucose metabolism to treat cancer have become a new research hotspot [7, 8]. Tumor cells can produce adenosine triphosphate (ATP) through glycolysis even under aerobic conditions. This phenomenon was first observed by Otto Warburg in the 1920s and is also called the “Warburg effect” [9]. The Warburg effect enables tumor cells to complete the transformation of energy supply through oxidative phosphorylation to glycolysis at normal oxygen concentrations [10]. Due to the low ATP production from aerobic glycolysis, tumor cells must have a relatively high glucose uptake capacity to meet their energy, biosynthetic and redox requirements. The conversion of glucose to glucose-6-phosphate (G6P, oxidative phosphorylation of glucose) is the first step in the glycolytic process and is the central biochemical event of cell metabolism. Four vertebrate hexokinase (HK) subtypes including glucokinase (GCK) and HK1-3 that catalyze this reaction have been extensively studied [11, 12]. Studies have shown that these HKs are closely related to the progression of a variety of tumors, including liver cancer [13], cervical cancer [14], colorectal cancer [15] and PCa [16], and therefore are promising targets for tumor therapy.

As a typical glycolytic enzyme of archaea, ADP-dependent glucokinase (ADPGK) has been found to be widely expressed in mammals and highly expressed in human hematopoietic and immune cells (such as T cells) [17]. ADPGK can utilize ADP to oxidize glucose and mediate the generation of oxidation signals [18]. Specifically, a study in T cells revealed that ADPGK can enhance the Warburg effect in T cells, but the specific mechanism of ADPGK in tumor cells is still unclear [19]. In addition, a study of lung and colon cancers manifested that knockdown of *ADPGK* expression in cells can reduce the colony formation ability of H460 cells but does not affect their glycolysis level [20]. Also, a study of Jurkat T cells proved that *ADPGK* gene knockout could promote the apoptosis of Jurkat T cells and endoplasmic reticulum stress and further inhibit the Warburg effect [21]. However, due to limited research, the biological behavior and mechanisms of ADPGK in tumor cells are still unclear.

The expression levels and prognostic roles of five HKs in PCa were systematically analyzed through bioinformatic analysis for the first time, and it was found that ADPGK may be a crucial gene contributing to PCa progression. The molecular mechanisms by which ADPGK promotes PCa progression were explored. We aimed to provide a new direction for the discovery of targets or biomarkers for PCa treatment.

## Methods

### Bioinformatic analysis

RNA-sequencing data from 33 tumors and matched normal tissue samples from patients were downloaded from The Cancer Genome Atlas (TCGA) dataset (https://genome-cancer.ucsc.edu/). For TCGA-PRAD, the clinical data were also downloaded. The level 3 transcripts per million formats were applied for subsequent analysis. The R package “ggplot2” was selected to analyze the differential expression among genes in matched tissue samples and the correlation between ADPGK and immune checkpoint gene expression. The R package “GSVA” was applied to analyze the differences between ADPGK expression and immune cell infiltration. The Kaplan‒Meier curves of overall survival (OS) and progression-free survival were drawn using the “survminer” package. The receiver operating characteristic (ROC) curve of selected genes for OS prediction was generated using the “pROC” and “ggplot2” packages. Binary logistic regression models were performed to analyze the associations of ADPGK expression and clinicopathological parameters.

### Online tools

Gene expression levels in normal and PCa tissues were analyzed using The Human Protein Atlas (https://www.proteinatlas.org/) [22]. Immunohistochemistry (IHC) images of genes of interest and the subcellular location of ADPGK were acquired. In addition, we also evaluated the association of ADPGK and cell type markers in the prostate. The TIMER algorithm (https://cistrome.shinyapps.io/timer/) was applied to analyze the correlation of ADPGK with prostate glandular and basal glandular cell markers, including prostatic acid phosphatase (ACPP), copine 4 (CPNE4), kallikrein-3 (KLK3), delta-like 2 (DLK2), fibronectin leucine rich transmembrane protein 3 (FLRT3) and keratin 5 (KRT5). The expression and correlation of ADPGK with aldolase C (ALDOC) were analyzed via cBioPortal (http://www.cbioportal.org/).

### Human samples and clinical study

PCa tissues and adjacent normal tissues were collected from PCa patients undergoing robot-assisted laparoscopic radical prostatectomy (RARP) at West China Hospital (Sichuan University, Chengdu, China) from June 2017 to December 2018. After excluding those who did not have complete clinical information and those who were lost during follow-ups, 45 PCa patients were finally included in this study. The study protocols were approved by the Ethical Review Committees of West China Hospital, Sichuan University (No. 2017–324) and written informed consent was acquired from all patients. PCa tumors and adjacent normal tissues were collected by urologists and identified by two independent pathologists after hematoxylin and eosin (HE) staining. Clinical information on age, weight and height, baseline prostate-specific antigen (PSA), neoadjuvant ADT, pathologic T (pT) stage, Gleason score (GS), extraprostatic extension (EPE), seminal vesicle invasion (SVI), perineural invasion (PNI), positive surgical margin (PSM), adjuvant radiotherapy, post RARP 3-month PSA and biochemical recurrence (BCR) were collected from their charts and reports. GS was determined according to the 2014 International Society of Urological Pathology grading system. Patient follow up was performed each month during the first three months post-RARP, every three months for the next two years, and once a year thereafter. BCR was defined as two consecutive increases of ≥ 0.2 ng/ml in PSA levels.

### Cell lines

WPMY-1, PC3, PC3M, 22Rv1, C4-2B and LNCaP cell lines were purchased from the American Type Culture Collection (ATCC, Manassas, Virginia, USA). PCa cell lines were cultured in RPMI 1640 medium (HyClone, Utah, USA) supplemented with 10% fetal bovine serum (FBS; Gibco, Australia) and 1% penicillin and streptomycin (HyClone) in a humidified incubator containing 5% CO_2_ at 37 °C. WPMY-1 cells were cultured in DMEM (HyClone) supplemented with 10% FBS (Gibco, Australia). PCa cell lines with ADPGK overexpression (ADPGK OE) and *ADPGK* knockdown (by sgRNA) were cultured in a complete culture medium with additional puromycin (2 μg/ml; KEHBIO, Beijing, China).

### Mouse model

The animal studies were authorized by the Animal Ethic Review Committees of the West China Hospital, China (No. 20170125252). All animal experiments were strictly implemented in compliance with the NIH Guide for the Care and Use of Laboratory Animals. Male nude BALB/c mice (18–20 g each) were purchased from Chengdu Dossy Experimental Animals Co., Ltd. (Chengdu, China). Ten mice were initially randomly assigned to two groups with five mice in each group. PC3 cells, including ADPGK OE and GFP cells, were prepared for the subcutaneous xenograft model. The concentration of cells in the two groups was determined and adjusted with saline to keep the number and volume of cells in the two groups consistent. A total of 5 × 10^6^ cells (200 μl per mouse) were injected subcutaneously into the right flank to construct a subcutaneous xenograft model. Tumor growth was monitored at 2-week intervals. The weight and tumor volume of the mice were measured every 3 d, and the formula for calculating volume was (length × width^2^)/2 [23]. The tumor sizes of the ADPGK OE group and GFP group were statistically analyzed using GraphPad Prism software (version 6.02; CA, USA). When there was a difference between the two groups, the mice were sacrificed, and the subcutaneous tumor, lung and liver of each mouse were harvested for Western blotting, immunofluorescence and HE staining. The antibodies used in this study are shown in Additional file 1: Table S1.

### Stable ADPGK overexpression cell line construction and screening

ADPGK overexpressing and ADPGK 6 × His-tag plasmids were constructed using lentiviruses purchased from PackGene Biotech (Guangzhou, China). After (2 – 3) × 10^5^ cells were seeded in a 6-well plate for 48 h, we replaced the culture medium in each well with 500 μl of lentivirus viral supernatant, 500 μl of fresh medium and 1 μg of polybrene. After 24 h, the culture medium was replaced with a complete medium. Twenty-four hours later, 2 μg/ml puromycin was added to each cell for screening. The cells were passaged in a new T24 flask with a complete medium (puromycin added) after they were overgrown.

### Small interfering RNA (siRNA) transfection

siRNAs targeting *ADPGK* and *ALDOC* were purchased from RiboBio (Guangzhou, China). The siRNA sequences are shown in Additional file 1: Table S2. The methods have been described previously [24].

### Clustered regularly interspaced short palindromic repeats (CRISPR)/Cas9-mediated gene editing

CRISPR/Cas9-mediated gene editing was described in our previous study [24]. Briefly, Cas9-expressing stable 22Rv1 cell lines were constructed, and puromycin screening was conducted. sgRNA oligos targeting *ADPGK* were designed, cloned and inserted into the pLentiCRISPR V2 plasmid (sequences listed in Additional file 1: Table S3). The harvested lentivirus was filtered (0.45 μm strainer), and 1 ml was added to the cell supernatant; 1000 × polybrene was also added.

### RNA extraction and real-time quantitative polymerase chain reaction (qPCR)

Total RNA was extracted by using an RNeasy Mini Kit (Qiagen, Texas, USA), and it was then used to synthesize first-strand cDNA using the Thermo Scientific RevertAid RT kit (Vilnius, Lithuania) with Oligo (dT)_18_. qPCR was performed with the QuantiNova SYBR Green PCR kit (Qiagen, Texas, USA), and the reactions were performed on a CFX96 Touch Real-Time PCR System (Bio-Rad, CA, USA). The specific settings and data analyses have been described previously [24]. The primer sequences were acquired from the PrimerBank website (https://pga.mgh.harvard.edu/primerbank/) and were synthesized by Sangon Biotech (Shanghai, China). The primers used in this study are shown in Additional file 1: Table S4.

### Cell counting kit-8 (CCK-8) assay

Cells were seeded in a 96-well plate (approximately 5 × 10^3^/well). When the cells in the 96-well plate grew to 80–90% confluence, they were cultured with fresh complete culture medium, followed by the addition of 10 μl of CCK-8 reagent (Dojindo Molecular Technologies, Rockville, USA). Acadesine (also known as AICAR; Selleck, TX, USA) was dissolved in a culture medium and diluted to 0.5 mmol/L before use [25]. Compound C (Selleck, TX, USA) was dissolved in DMSO and diluted in a culture medium to 10 μmol/L before use [26]. The ADPGK inhibitor 8-Bromo-AMP [MedChemExpress (MCE), NJ, USA] was dissolved in phosphate-buffered saline (PBS) [27]. Cells were incubated at 37 °C in the dark for 2 h. Then, the plate was placed in the EonTM Microplate Reader (BioTek, VT, USA) to measure the absorbance at 450 nm.

### 5-ethynyl-2'-deoxyuridine (EdU) assay

PCa cells were digested and seeded in 96-well plates at a density of 3 × 10^3^ cells per well. When the cells in the 96-well plate grew to 70–80%, 50 μmol/L EdU (RiboBio Biotechnology, Guangzhou, China) was then added to each well and incubated for 2 h, after which the cells were washed twice using PBS and then fixed with 4% paraformaldehyde for 30 min. The cells were incubated with glycine solution for 5 min at room temperature, after which 1 × Apollo dye solution was added, and cells were incubated in a shaker at room temperature and kept away from light for 30 min. DNA staining was performed after the cells were washed 3 times (10 min each) using 100 μl of osmotic agent. Images were obtained using an OBSERVER D1/AX10 cam heat release capacity (HRC) microscope (Zeiss, Oberkochen, Germany). The Cell-Light EdU Apollo643 In Vitro Kit (RiboBio Biotechnology) was used to perform the EdU flow cytometry test.

### Colony formation assay

Cells were seeded in 6-well plates at 1000 cells/well for 10–14 d. The cells were washed with PBS and fixed with cold methanol for 20 min. Subsequently, the cells were stained with crystal violet (Beyotime, Shanghai, China) for 15–20 min. Finally, the cells were washed and imaged using a Celigo Imaging Cytometer.

### Wound healing assay

Cells were seeded in a 6-well plate after digestion, collected by centrifugation and counted at 3 × 10^5^ cells per well. When the cells grew to approximately 80–90% confluence, 3 vertical parallel lines were drawn with a 10 μl sterile spear point in each well. Each well was washed twice with PBS to wash off the suspended dead cells, and 2 ml of fresh medium containing 1% FBS was added to each well. Images were taken under an inverted fluorescence Zeiss OBSERVER D1/AX10 CAM HRC microscope (Zeiss, Oberkochen, Germany), and the position of each well was recorded. After the photo was taken, the 6-well plates were placed in the cell incubator for further incubation. Twenty-four hours later, the wells were photographed again at the same position.

### Transwell assay

By using previously described methods [24], Transwell assays were performed, and the chambers were imaged under an inverted fluorescence OBSERVER D1/AX10 cam HRC microscope (Zeiss, Oberkochen, Germany). The number of transferred cells was analyzed by ImageJ software (version 1.48; National Institutes of Health, USA).

### Co-immunoprecipitation (Co-IP)

Co-IP experiments were performed using a Pierce Co-IP kit (Thermo, IL, USA). Briefly, 10 μg of His-tag and IgG antibodies were added to the resins and incubated on a mixer for 120 min at room temperature. Afterward, coupling buffer, quenching buffer and sodium cyanoborohydride solution were added in sequence, and the resins were washed using wash solutions. Cells in 10 × 10 cm dishes were collected by adding 500 μl IP lysis/wash buffer. Next, the cell lysates were adjusted to a 1 μg/μl concentration for subsequent use. The cell lysate was precleared using the control agarose resin at 4 °C for 30 min. Next, the precleared lysate was added to the antibody-coupled resin and incubated at 4 °C overnight. Afterward, the resin was washed, and an elution buffer was added to obtain the final IP samples. Loading buffer (5 ×) was added to the IP samples, and the samples were heated at 100 °C for 10 min for subsequent Western blotting analysis.

### Western blotting analysis

Proteins were extracted using RIPA lysis buffer (Thermo Scientific, IL, USA) supplemented with protease inhibitor (Roche, Mannheim, Germany) and phosphatase inhibitor (Thermo Scientific, IL, USA) cocktails. A BCA Protein Assay Kit (Beyotime Biotechnology, Shanghai, China) was used to assess the protein concentration. After the protein was denatured at 100 °C, a PAGE Gel Fast Preparation Kit (Epizyme, Shanghai, China) was applied to perform sodium dodecyl sulfate‐polyacrylamide gel electrophoresis (SDS‒PAGE), and the proteins were transferred onto a PVDF membrane (Millipore, MA, USA) at 300 mA for 65 min. Afterward, the membranes were blocked for 60–90 min in 5% skim milk powder and incubated with primary antibodies overnight at 4 °C. After washing 3 times using 1 × TBST, the cells were incubated with secondary antibodies for 1 h. Immunoreactivity was visualized using enhanced chemiluminescent (ECL) chromogenic substrate (Millipore, MA, USA). The membranes were finally detected by using a ChemiDoc MP Imager System (Bio-Rad, CA, USA).

### IHC

Specimens were fixed in 4% paraformaldehyde at room temperature and embedded in paraffin. Then, the tissues were cut into 4 μm thick sections. Subsequently, we dewaxed, hydrated and incubated the tissues with antibodies (ADPGK and ALDOC) overnight at 4 °C. After incubation with the corresponding secondary antibodies, the sections were stained with diaminobenzidine and reverse stained with hematoxylin. Two independent pathologists independently scored the positive IHC staining. ADPGK or ALDOC low and high expression was defined as 0–50% positive cells and 50–100% positive cells, respectively.

### Immunofluorescence and HE staining

Tissues were stained by immunofluorescence using ADPGK antibody. The tissues were dehydrated and implanted in optimal cutting temperature (Sakura, CA, USA) compound after being treated with 10%, 20% and 30% sucrose solution at 4 °C overnight. Then, the embedded tissues were sectioned with a frozen microtome. The slices were sealed in blocking fluid (5% BSA + 10% serum in 1 × PBS) at room temperature for 1 h and sealed in ADPGK antibody at 4 °C overnight. After the slides were washed with PBS, they were blocked with a secondary antibody at room temperature and kept in the dark for 1 h. After DAPI staining and DDW rinse application, we sealed the slides with an anti-quench agent and placed them in a fume hood overnight away from light. The liver and lung tissues of mice were fixed in 4% formaldehyde and embedded in paraffin. After cutting the tissues into 4 μm thick sections, the tissue sections were deparaffinized and rehydrated. HE staining was carried out for the next process. Cells were fixed with 4% paraformaldehyde, washed with PBS, processed with 0.5% Triton X-100, and blocked with goat serum for 30 min. ALDOC/His-tag primary antibodies were added overnight incubation at 4 °C. Secondary antibodies [Alexa Fluor™ 594 goat anti-mouse IgG (H + L) and Alexa Fluor™ 488 goat anti-rabbit IgG (H + L)] were added accordingly and incubated for 1 h at room temperature. After DAPI staining for 5 min, images were obtained under a Nikon A1RMP + laser scanning confocal microscope (Nikon, Japan) and an AX10 imager A2/AX10 cam HRC (Zeiss, Oberkochen, Germany).

### Quantitative proteomics

For quantitative proteomics, PC3 cell samples, including ADPGK OE and GFP cells (*n* = 3), were sonicated and centrifuged to obtain proteins. After trypsin digestion of the protein solutions, tandem mass tags (TMT) and isobaric tags for relative absolute quantitation (iTRAQ) labeling were performed, and the tryptic peptides were subjected to liquid chromatograph-mass spectrometer (LC–MS)/MS analysis. The resulting MS/MS data were processed using the MaxQuant search engine (v.1.5.2.8). The mass tolerance for precursor ions was set as 20 ppm in the first search and 5 ppm in the main search, and the mass tolerance for fragment ions was set as 0.02 Da. Carbamidomethyl on Cys was specified as a fixed modification, and acetylation and oxidation on Met were specified as variable modifications. The false discovery rate was adjusted to < 1%, and the minimum score for modified peptides was set to > 40. The Gene Ontology (GO) annotated proteome data were derived from the UniProt-GOA database (http://www.ebi.ac.uk/GOA/). Kyoto Encyclopedia of Genes and Genomes (KEGG) pathway annotation was performed using the KEGG online service tool KAAS.

### Metabolomics

For metabolomics, PC3 cell samples from the two groups (ADPGK OE cells and GFP cells; *n* = 3) were prepared and stored at –80 °C. Quality control samples were pooled by mixing cells (10 μl) from the subjects to ensure the data quality for metabolic profiling. UPLC-Q-TOF/MS was performed on an Agilent 1290 Infinity with a 6530 Q/TOF-MS system (Agilent Technologies, USA). The next analysis was performed on a triple quadrupole LC-MS/MS system (LCMS8050, Shimadzu, Japan).

### Extracellular acidification rate (ECAR) and oxygen consumption rate (OCR) measurements

The ECAR test was carried out using the Agilent Seahorse XFe24 Analyzer (DE, USA). Briefly, cells were harvested and seeded in 24-well Seahorse XF Cell Culture Microplates (LNCaP and 22Rv1, 3 × 10^4^ cells per well; PC3 and PC3M: 2 × 10^4^ cells per well). For LNCaP and 22Rv1 cells, siRNA transfection was performed on the second day, and 0.5 mmol/L AICAR was added and incubated for 24 h. For ADPGK OE cells, 10 μmol/L Compound C was added and incubated for 24 h. Then, XF assay media was prepared, which was supplemented with Seahorse XF Base Medium (Seahorse Bioscience, CA, USA) and 2 mmol/L L-glutamine (Life Tech, CA, USA). The assay medium was warmed to 37 °C, and the pH was adjusted to 7.4. XF assay media was added to the 24-well microplate and incubated in a CO_2_-free incubator at 37 °C for 60 min. Afterward, 10 mmol/L glucose (Sigma-Aldrich, Shanghai, China), 1 μmol/L oligomycin (Sigma-Aldrich, Shanghai, China), and 50 mmol/L 2-deoxy-glucose (Sigma-Aldrich, Shanghai, China) were added to the microplate in sequence. The OCR test was performed at 37 °C after sequential supplementation with 4 mmol/L ADP (Sigma-Aldrich, Shanghai, China), 2.5 μg/ml oligomycin (MCE, NJ, USA), 4 μmol/L fluorocarbonyl cyanide phenylhydrazone (FCCP; MCE, NJ, USA), and 4 μmol/L antimycin A (Sigma-Aldrich, Shanghai, China). All readings are normalized to cell number.

### Statistical analysis

Error bars indicate the mean ± standard deviation unless otherwise indicated. Student’s *t*-test and one-way analysis of variance were used to analyze the differences between groups. GraphPad Prism 7.0 was used to perform statistical analysis and graphics plotting for in vitro and in vivo studies. Statistical significance is indicated by *P < 0.05, **P < 0.01, ***P < 0.001, and ****P < 0.0001. SPSS (version 15.0, IBM corporation) was used to analyze clinical data, and Kaplan‒Meier curves with the log-rank test were applied for survival analysis. Univariate and multivariate Cox regression analyses were performed to probe the association of ADPGK expression with BCR. Variables with a *P-*value < 0.1 in the univariate analysis were included in the multivariate Cox analysis. The results are presented by R version 3.6.1.

## Results

### Function, expression and clinical outcomes of genes involved in the oxidative phosphorylation of glucose

To obtain genes involved in the oxidative phosphorylation of glucose, the metabolic atlas (https://metabolicatlas.org/) was searched and five related genes: *GCK*, *HK1*, *HK2*, *HK3* and *ADPGK* were obtained. Their roles in the conversion of glucose to G6P are shown in Additional file 1: Fig. S1a. The mRNA expression of these genes in TCGA-PRAD samples was determined, and in both non-paired (Additional file 1: Fig. S1b) and paired samples (Additional file 1: Fig. S1c), *HK2*, *HK3* and *ADPGK* were overexpressed, while *GCK* and *HK1* were downregulated in tumor samples compared with normal tissues. The IHC results of The Human Protein Atlas displayed that HK1 and HK3 were lowly expressed in PCa. Compared with normal prostate tissue, ADPGK was significantly highly expressed in PCa tissues (Additional file 1: Fig. S1d). Next, the ROC curves were used to analyze their sensitivities and specificities in predicting clinical outcomes. The results showed that ADPGK had a higher area under the curve (AUC) for predicting high T stage (≥ 3, AUC = 0.603) and GS (≥ 8, AUC = 0.609) than GCK, HK1, HK2 and HK3 (Additional file 1: Fig. S1e). Moreover, ADPGK manifested the highest AUC for predicting 3-, 8- and 10-year OS compared with other HKs (Additional file 1: Fig. S1e). Survival curves revealed that only ADPGK was a negative prognostic factor for OS in PCa (log-rank *P* = 0.029, Additional file 1: Fig. S1f). Overall, these results suggested that ADPGK might play a fundamental role in PCa progression.

### Cell type enrichment, subgroup prognosis and immune correlation of ADPGK in PRAD

The association of ADPGK with prostate tissue markers for characterizing ADPGK enrichment in prostate tissue cell types was analyzed, and ADPGK was significantly correlated with prostate glandular cell markers (ACP3, CPNE4, KLK3, DLK2, FLRT3, and KRT5, Additional file 1: Fig. S2a). In PCa, a significant positive correlation between ADPGK and ACP3 (also known as ACPP), CPNE4, KLK3 was established (Additional file 1: Fig. S2b). Next, by performing pan-cancer analysis, high expression of ADPGK was observed in PRAD and other cancer types, such as uterine corpus endometrial carcinoma (UCEC), stomach adenocarcinoma (STAD), and rectum adenocarcinoma (READ) (Additional file 1: Fig. S2c). Furthermore, the prognostic role of ADPGK was more significant in patients with age > 60 years, GS ≥ 8 and T stage ≥ 3 by subgroup analysis (Additional file 1: Fig. S2d). It also suggested an association between ADPGK and PCa immune status. The results showed that high ADPGK expression was associated with significantly decreased immune cell infiltration in PCa (Additional file 1: Fig. S3a). A negative correlation between ADPGK and tumor mutation burden in PCa was also observed, albeit the coefficients are small (Additional file 1: Fig. S3b). Moreover, ADPGK was negatively correlated with immune checkpoint-related genes (Additional file 1: Fig. S3c), which suggests that targeting ADPGK might activate the immune response in PCa.

### High ADPGK expression is associated with poor outcomes in PRAD patients

**Fig. 1:**
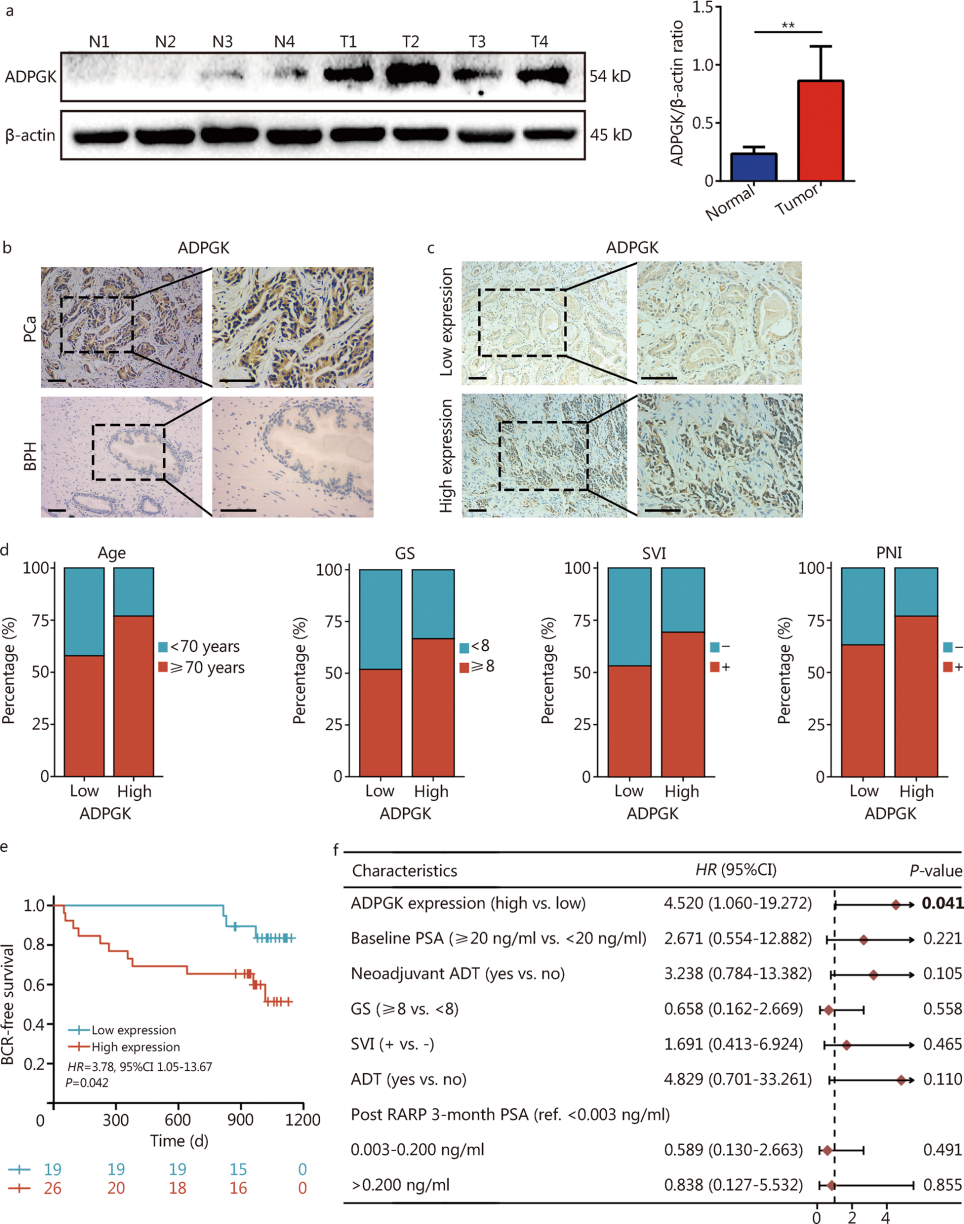
High ADPGK expression is associated with poor oncologic outcomes. **a** Validation of ADPGK expression in clinical PCa samples by Western blotting (*n* = 4). **b** ADPGK expression status in the clinical samples assessed by immunohistochemistry (IHC). Scale bar = 100 μm. **c** PCa samples were divided into low and high ADPGK expression groups according to the IHC results. Scale bar = 100 μm. **d** Associations of ADPGK with different clinical features. **e** Kaplan‒Meier curves showing the association of ADPGK with BCR in 45 patients. **f** Multivariate Cox regression analyses of the relationship between parameters with BCR. Data are presented as the mean ± SD. **P < 0.01. ADPGK ADP-dependent glucokinase, N normal, T tumor, PCa prostate cancer, BPH benign prostate hyperplasia, GS Gleason score, SVI seminal vesicle invasion, PNI perineural invasion, HR hazard ratio, SD standard deviation, BCR biochemical recurrence, ADT androgen deprivation therapy, PSA prostate specific antigen, RARP robot-assisted laparoscopic radical prostatectomy

ADPGK is highly expressed in PCa patients compared with normal prostate tissues (Fig. 1a). In addition, by performing an IHC assay, the team has observed that ADPGK expression was also higher in PCa tissues than in benign prostate hyperplasia (BPH) tissues (Fig. 1b). Subsequently, PCa samples were divided into an ADPGK high expression group and low expression group according to the IHC results (Fig. 1c). The baseline characteristics of the included patients are shown in Table 1. Among the 45 PCa patients, there were 26 patients in the ADPGK high expression group, and 19 patients in the ADPGK low expression group. The median age was 64 years, and the median follow-up time was 32.6 months. Twenty-seven (60.0%) patients had GS lower than 8, and 18 (40.0%) patients had GS higher than 8. There were no significant differences between the two groups in baseline parameters (all *P* > 0.05), except for neoadjuvant ADT (*P* = 0.024). The ADPGK high expression group had a higher GS, greater SVI and PNI than the ADPGK low expression group, although this difference was not statistically significant (Fig. 1d). The data showed that high ADPGK expression was associated with short BCR-free survival (log-rank *P* = 0.029; Fig. 1e). Univariate analysis revealed that neoadjuvant ADT (*P* = 0.003), ADPGK expression (*P* = 0.042), GS (*P* = 0.043), SVI (*P* = 0.007) and ADT (*P* = 0.017) were risk factors for BCR (Additional file 1: Table S5). The multivariate Cox models verified that ADPGK could serve as an independent factor in predicting the BCR of PCa patients (*P* = 0.041; Fig. 1f).

**Table 1:** Characteristics of patients in the present study [*n*(%)]

Variables	Total (*n* = 45)	ADPGK low expression (*n* = 19, 42.2%)	ADPGK high expression (*n* = 26, 57.8%)	*P*
Age (years)				0.206
< 70	14 (31.1)	8 (42.1)	6 (23.1)	
≥ 70	31 (68.9)	11 (57.9)	20 (76.9)	
BMI (kg/m^2^)				1.000
< 25	30 (66.7)	13 (68.4)	17 (65.4)	
≥ 25	15 (33.3)	6 (31.6)	9 (34.6)	
Baseline PSA (ng/ml)				0.764
< 20	21 (46.7)	8 (42.1)	13 (50.0)	
≥ 20	24 (53.3)	11 (57.9)	13 (50.0)	
Neoadjuvant ADT				0.024
No	32 (71.1)	17 (89.5)	15 (57.7)	
Yes	13 (28.9)	2 (10.5)	11 (42.3)	
pT stage				1.000
< pT3	21 (46.7)	9 (47.4)	12 (46.2)	
≥ pT3	24 (53.3)	10 (52.6)	14 (53.8)	
GS				0.371
< 8	27 (60.0)	13 (68.4)	14 (53.8)	
≥ 8	18 (40.0)	6 (31.6)	12 (46.2)	
EPE				1.000
−	21 (46.7)	9 (47.4)	12 (46.2)	
+	24 (53.3)	10 (52.6)	14 (53.8)	
SVI				0.507
−	32 (71.1)	15 (78.9)	17 (65.4)	
+	13 (28.9)	4 (21.1)	9 (34.6)	
PNI				0.341
−	13 (28.9)	7 (36.8)	6 (23.1)	
+	32 (71.1)	12 (63.2)	20 (76.9)	
PSM				0.734
−	12 (26.7)	6 (31.6)	6 (23.1)	
+	33 (73.3)	13 (68.4)	20 (76.9)	
ADT				0.770
No	20 (44.4)	9 (47.4)	11 (42.3)	
Yes	25 (55.6)	10 (52.6)	15 (57.7)	
Adjuvant radiotherapy				0.371
No	27 (60.0)	13 (68.4)	14 (53.8)	
Yes	18 (40.0)	6 (31.6)	12 (46.2)	
Post RARP 3-month-PSA (ng/ml)				0.764
< 0.003	10 (22.2)	5 (26.3)	5 (19.2)	
0.003–0.200	26 (57.8)	11 (57.9)	15 (57.7)	
> 0.200	9 (20.0)	3 (15.8)	6 (23.1)	

*ADPGK* ADP-dependent glucokinase, *BMI* body mass index, *PSA* prostate specific antigen, *ADT* androgen deprivation therapy, *pT* pathologic T, *GS* Gleason score, *EPE* extraprostatic extension, *SVI* seminal vesicle invasion, *PNI* perineural invasion, *PSM* positive surgical margin

### ADPGK promotes PCa cell proliferation and migration in vitro

**Fig. 2:**
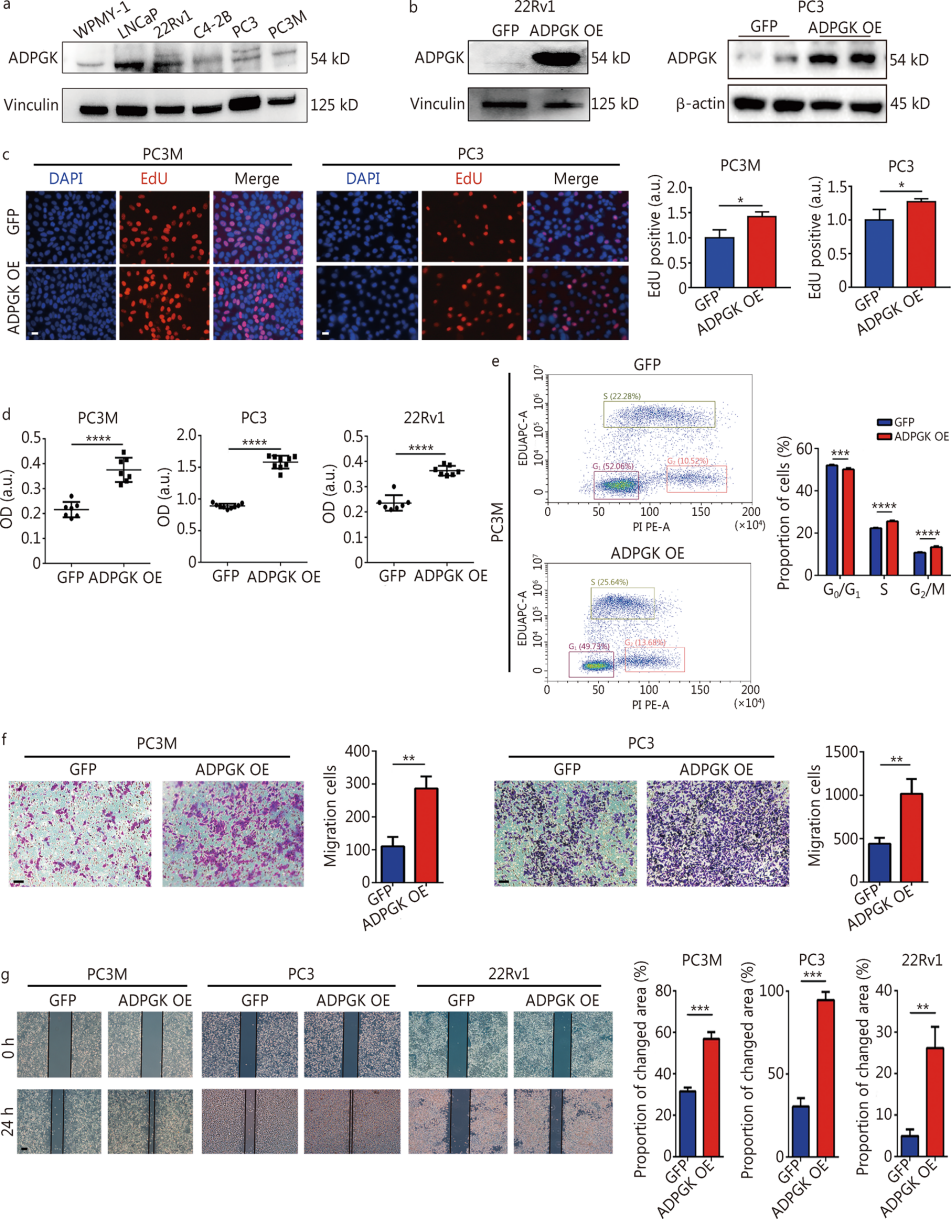
ADPGK overexpression promotes PCa cell proliferation and migration in vitro. **a** ADPGK protein expression across prostate cell lines validated by Western blotting. **b** Western blotting results showed the stable overexpression of ADPGK in 22Rv1 and PC3 cells after lentivirus transfection. **c** Cell proliferation was measured by EdU assays (*n* = 3). Scale bar = 20 μm. **d** Cell viability was assessed by CCK-8 assays (*n* = 3). **e** Cell proliferation was measured by EdU flow cytometry analysis (*n* = 3).** f** Cell migration was assessed by Transwell assays (*n* = 3). Scale bar = 50 μm. **g** Cell migration was assessed by wound healing assays (*n* = 3). Scale bar = 200 μm. Data are presented as the mean ± SD. *P < 0.05, **P < 0.01, ***P < 0.001, ****P < 0.0001. ADPGK ADP-dependent glucokinase, PCa prostate cancer, SD standard deviation, OE overexpression, OD optical density, a.u. artificial unit

Evaluation was carried out on ADPGK protein expression in several prostate cell lines. Compared with the normal prostate cell line WPMY-1 [28], the expression of ADPGK was relatively higher in LNCaP and 22Rv1 cells (Fig. 2a). Stable ADPGK overexpressing cell lines in three PCa cell lines were constructed by lentivirus transfection. The qPCR results showed that *ADPGK* was markedly overexpressed in the PC3M, PC3 and 22Rv1 cell lines (Additional file 1: Fig. S4). They were also validated by Western blotting (Fig. 2b). The impact of ADPGK on PCa cell proliferation ability was assessed through the EdU assay, which displayed that ADPGK could promote the proliferation of PCa cells (Fig. 2c). The CCK-8 assay demonstrated that the cell proliferation activities of PC3M, PC3, and 22Rv1 cells were all significantly enhanced compared with the control group (Fig. 2d). In addition, S phase and G_2_/M phase were accelerated by ADPGK overexpression shown by EdU flow cytometry (Fig. 2e). Transwell assays and wound healing assays demonstrated that the overexpression of ADPGK significantly promoted PCa cell migration (Fig. 2f, g).

### *ADPGK* silencing suppresses PCa progression in vitro

**Fig. 3:**
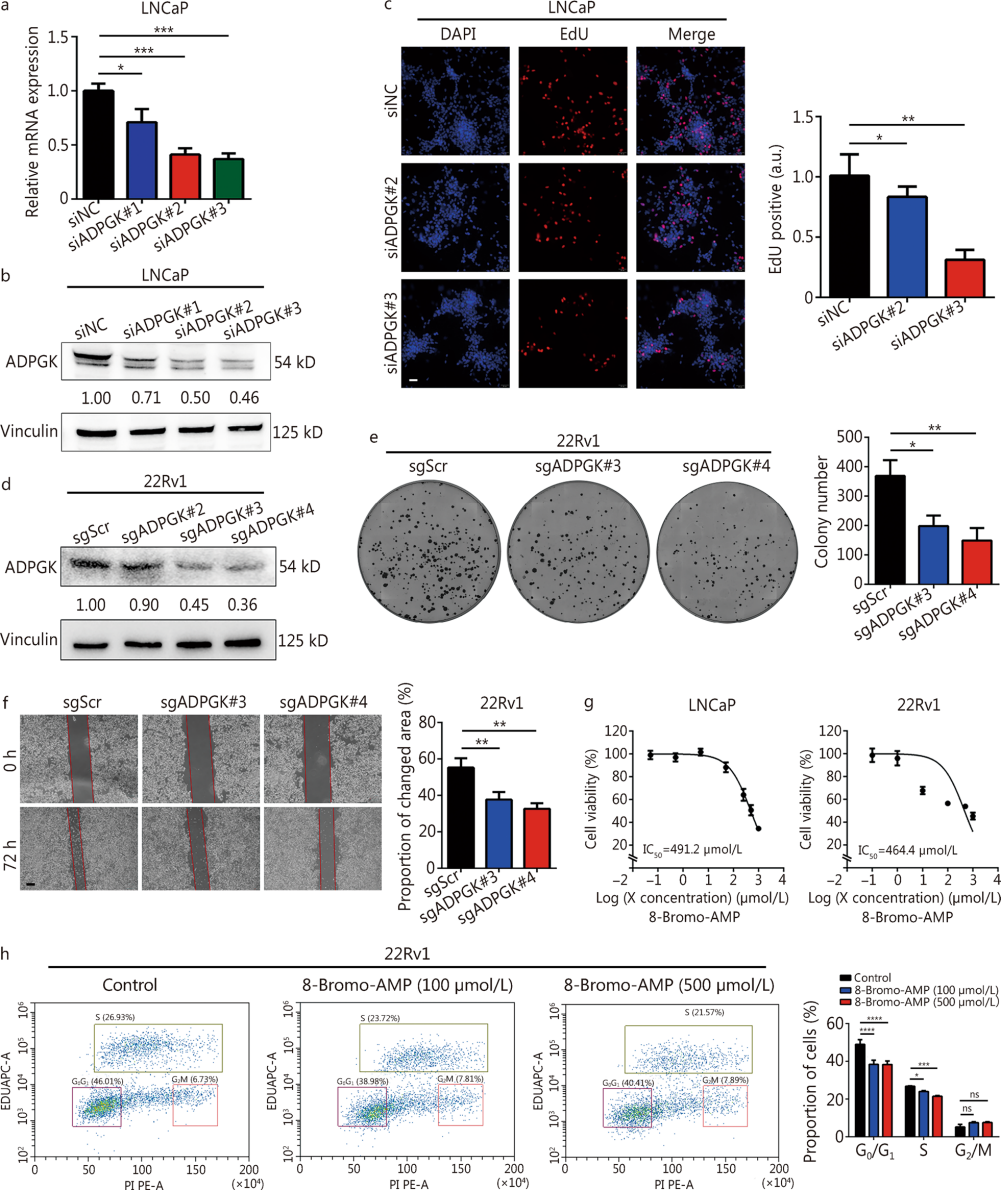
Inhibition of ADPGK suppresses PCa progression in vitro. **a** RNAi**-**mediated *ADPGK* knockdown was validated by qPCR. **b** RNAi**-**mediated *ADPGK* knockdown was validated by Western blotting. **c** Cell proliferation was measured by EdU assay after *ADPGK* knockdown (*n* = 3). Scale bar = 50 μm. **d** CRISPR/Cas9-mediated *ADPGK* knockdown was validated by Western blotting. **e** The effect of *ADPGK* knockdown on cell proliferation was validated in a colony formation assay (*n* = 3). **f** The effect of *ADPGK* knockdown on PCa cell migration ability was validated in a wound healing assay (*n* = 3). Scale bar = 200 μm. **g** A CCK-8 assay was performed to evaluate the effect of an ADPGK inhibitor (8-Bromo-AMP) on PCa cells (*n* = 5). **h** An EdU flow cytometric assay was performed in 22Rv1 cells using different concentrations of 8-Bromo-AMP (*n* = 3). Data are presented as the mean ± SD. *P < 0.05, **P < 0.01, ***P < 0.001, ****P < 0.0001, ns non-significant. Scr scramble, PCa prostate cancer, SD standard deviation, NC negative control, ADPGK ADP-dependent glucokinase, qPCR quantitative polymerase chain reaction, CRISPR clustered regularly interspaced short palindromic repeats, a.u. artificial unit

siRNA-mediated *ADPGK* knockdown was performed in LNCaP cells. The results showed that all three siRNAs significantly reduced the *ADPGK* mRNA (Fig. 3a) and protein (Fig. 3b) levels compared with siNC. *ADPGK* knockdown significantly inhibited PCa proliferation (Fig. 3c, Additional file 1: Fig. S5). In addition, CRISPR/Cas9-mediated *ADPGK* knockdown was performed in 22Rv1 cells (Fig. 3d). *ADPGK* silencing suppressed PCa cell colony formation (Fig. 3e) and migration ability (Fig. 3f). Furthermore, the ADPGK inhibitor 8-Bromo-AMP had cytotoxic effects on LNCaP and 22Rv1 cells (Fig. 3g). 8-Bromo-AMP significantly suppressed the PCa cell cycle (mainly in the S phase, Fig. 3h). The above results supported ADPGK as a driving factor for PCa progression.

### ADPGK accelerates PCa growth and liver metastasis in vivo

**Fig. 4:**
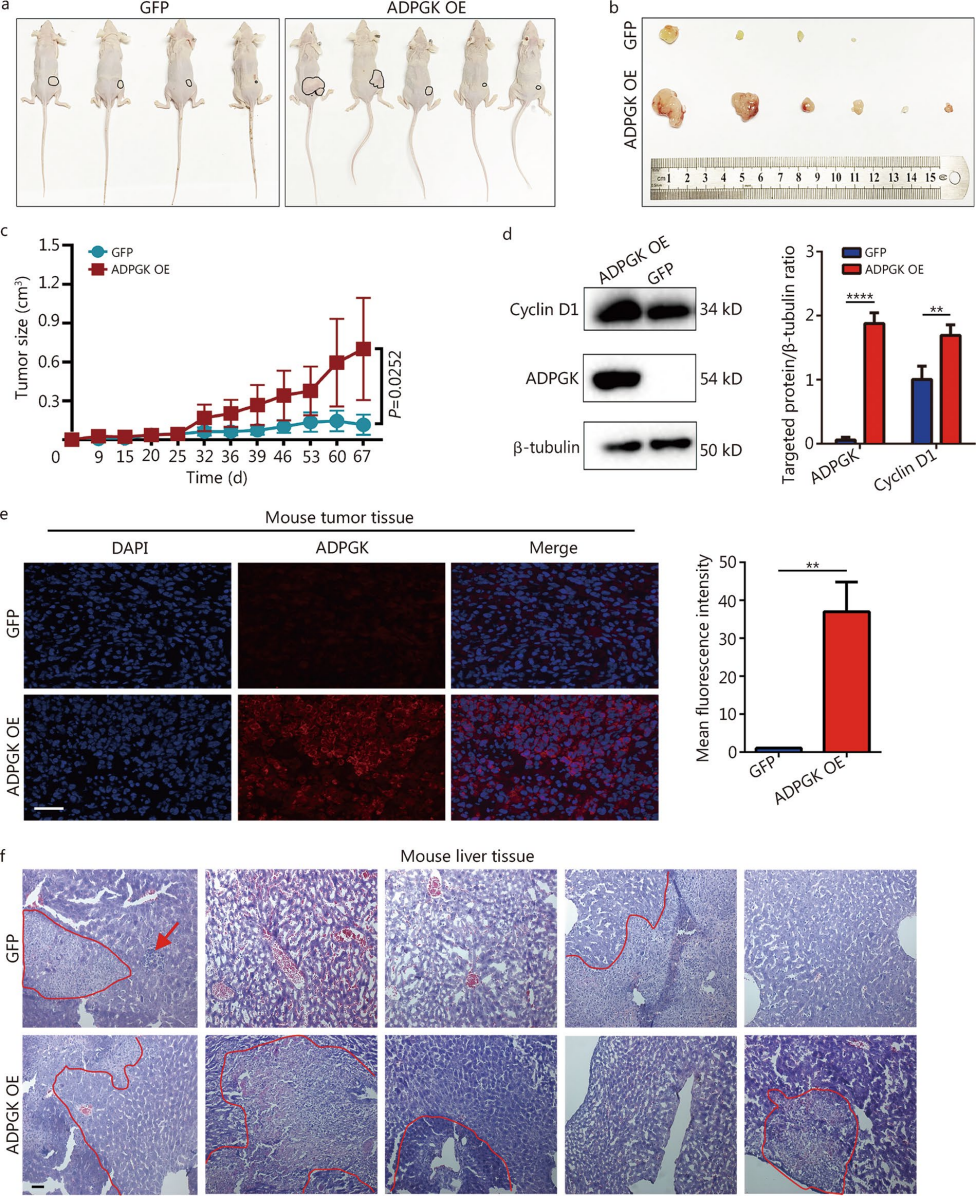
ADPGK promotes PCa growth and liver metastasis in vivo. **a** Tumor-bearing mice in two groups (the tumor of one mouse in the control group did not arise). **b** Tumor size of the two groups (one mouse in the ADPGK OE group grew 2 subcutaneous tumors, contributing to the final 6 tumors shown in the ADPGK OE group). **c** Changes in tumor growth size over time. **d** Cyclin D1 and ADPGK protein expression in xenograft tissues assessed by Western blotting (*n* = 3). **e** Verification of tumor tissues from the xenograft mice by immunofluorescence with an ADPGK antibody (*n* = 3). Scale bar = 50 μm. **f** Metastatic foci of the liver in the tumor-bearing mice by hematoxylin and eosin (HE) staining (circled in red line). Scale bar = 50 μm. Data are presented as the mean ± SD. **P < 0.01, ****P < 0.0001. PCa prostate cancer, ADPGK ADP-dependent glucokinase, SD standard deviation, NC negative control, OE overexpression

The results revealed that the tumor volume of the ADPGK OE group was significantly larger than that of the control group (Fig. 4a, b), and the growth curve revealed that ADPGK overexpression could significantly promote the growth of xenografts (Fig. 4c), with a significant difference (*P* = 0.0252). The Western blotting results confirmed the overexpression of ADPGK in the experimental group, which also showed increased expression of cyclin D1 (Fig. 4d). Immunofluorescence assays of subcutaneous tumor tissues revealed that ADPGK was also significantly upregulated in the ADPGK OE group (Fig. 4e). Furthermore, HE staining of mouse liver tissues proved that the number and size of the metastatic foci in the ADPGK OE group were higher than those in the control group, indicating that the overexpression of ADPGK promoted the liver metastasis of PCa (Fig. 4f). The HE staining of mouse lung tissues did not exhibit show metastatic lesions (Additional file 1: Fig. S6).

### ADPGK controls PCa metabolic fitness

**Fig. 5:**
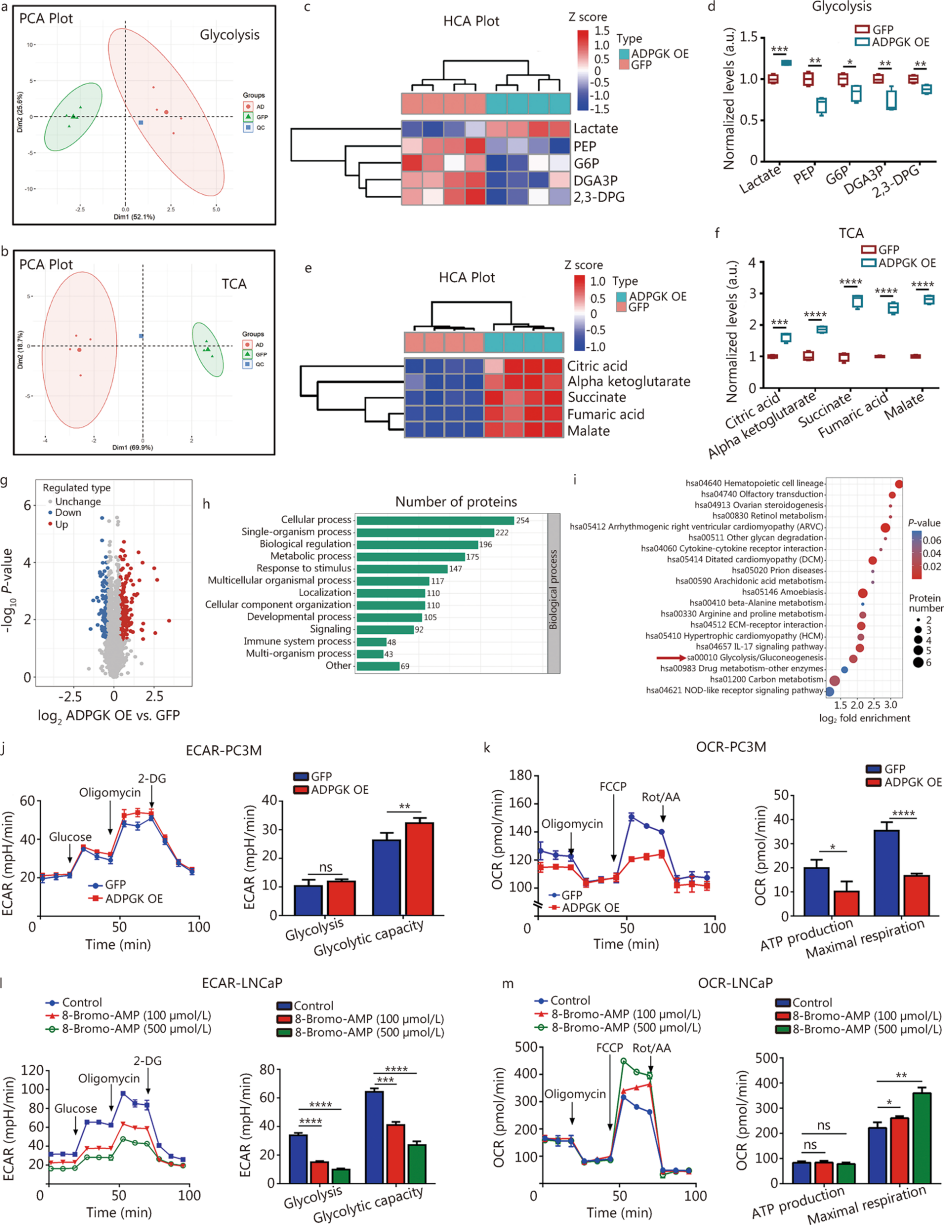
ADPGK controls PCa metabolic fitness. Principal component analysis (PCA) of the metabolomics of glycolysis (**a**) and the TCA cycle (**b**). **c** HCA plot from metabolomics of glycolysis. **d** Normalized levels of glycolytic products after ADPGK overexpression in PC3 cells. **e** HCA plot from metabolomics of TCA products. **f** Normalized levels of TCA products after ADPGK overexpression in PC3 cells. **g** A volcano plot from quantitative proteomics showed the differentially expressed proteins identified by quantitative proteomics (*P-*value < 0.05; |log_2_ fold change|> 1.3). GO (**h**) and KEGG (**i**) enrichment analysis of upregulated genes. ECAR (**j**) and OCR (**k**) tests in PC3M cells (*n* = 3). ECAR (**l**) and OCR (**m**) tests were performed in LNCaP cells after incubation with 8-Bromo-AMP for 72 h (*n* = 4). Data are presented as the mean ± SD. *P < 0.05, **P < 0.01, ***P < 0.001, ****P < 0.0001, ns non-significant. PCa prostate cancer, ADPGK ADP-dependent glucokinase, PEP phosphoenolpyruvate, G6P glucose-6-phosphate, DGA3P 3-phosphoglyceraldehyde, 2,3-DPG 2,3-diphosphoglycerate, TCA tricarboxylic acid, HCA hierarchical cluster analysis, GO Gene Ontology, KEGG Kyoto Encyclopedia of Genes and Genomes, ECAR extracellular acidification rate, OCR oxygen consumption rate, 2-DG 2-deoxy-glucose, FCCP fluorocarbonyl cyanide phenylhydrazone, Rot/AA rotenone/antimycin A, SD standard deviation, NC negative control, OE overexpression, a.u. artificial unit

Metabolomics was used to further reveal the potential mechanism related to ADPGK. Principal component analysis showed that the quality control of the samples was satisfactory (Fig. 5a, b). The results indicated that ADPGK overexpression decreased the expression of glycolytic pathway intermediates, including phosphoenolpyruvate, G6P, 3-phosphoglyceraldehyde and 2,3-diphosphoglycerate, and increased lactate production (Fig. 5c, d). In addition, metabolomics revealed that ADPGK overexpression elevated the levels of the tricarboxylic acid (TCA)-related products such as citric acid, alpha ketoglutarate, succinate, fumaric acid and malate (Fig. 5e, f). To further investigate the molecular mechanism of ADPGK in PCa progression, quantitative proteomics were conducted. When setting the threshold as *P-*value < 0.05 and |log_2_ fold change|> 1.3, there were 170 upregulated proteins and 121 downregulated proteins (Fig. 5g). Subsequent GO and KEGG enrichment analyses revealed that these genes were mainly enriched in metabolic processes (Fig. 5h) and glycolysis (Fig. 5i). To validate whether ADPGK could regulate metabolic processes, ECAR and OCR assays were carried out. The assays have shown ADPGK overexpression improved glycolytic capacity (Fig. 5j) and decreased ATP production and maximal respiration (Fig. 5k). In addition, inhibition of ADPGK with 8-Bromo-AMP decreased the glycolysis and glycolytic capacity (Fig. 5l), and increased the maximal respiration (Fig. 5m). These results indicated that ADPGK might participate in regulating PCa metabolic fitness.

### ADPGK regulates PCa glycolysis through AMPK phosphorylation

**Fig. 6:**
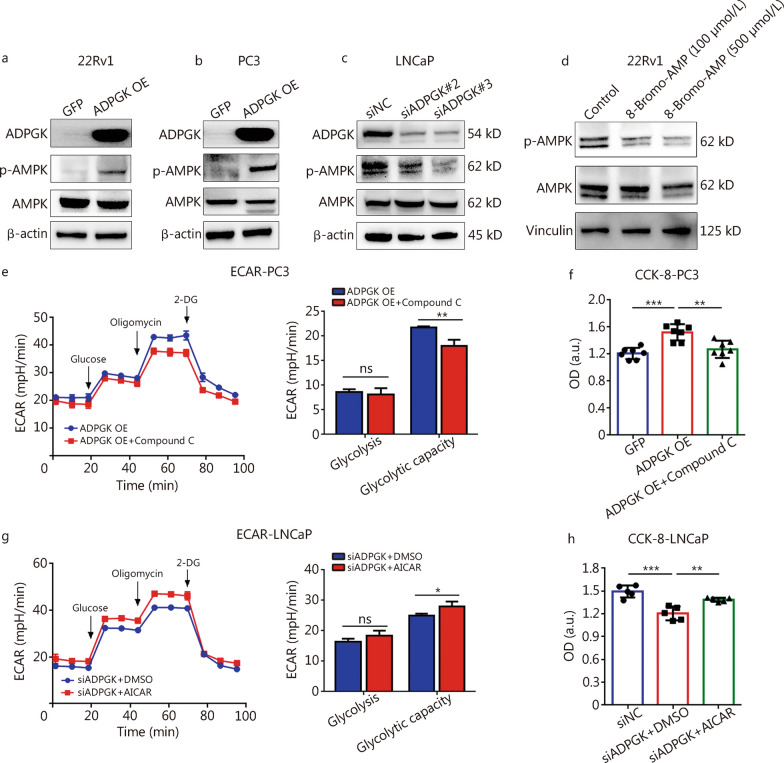
ADPGK regulates PCa glycolysis through AMPK phosphorylation. Effect of ADPGK overexpression on AMPK and p-AMPK protein expression levels in 22Rv1 (**a**) and PC3 (**b**) cells. **c** Effect of *ADPGK* knockdown by siRNA on AMPK and p-AMPK protein expression levels in LNCaP cells. **d** Effect of the ADPGK inhibitor 8-Bromo-AMP on AMPK and p-AMPK protein expression levels in 22Rv1 cells. **e** ECAR test in ADPGK OE and ADPGK OE + 10 μmol/L Compound C (a type of AMPK inhibitor) in PC3 cells (*n* = 4). **f** CCK-8 assay showed the cell viability changes after ADPGK overexpression and Compound C addition (10 μmol/L) (*n* = 7). **g** ECAR results of siADPGK#3 and siADPGK#3 + 0.5 mmol/L acadesine (AICAR, a type of AMPK agonist) in LNCaP cells (*n* = 3). **h** CCK-8 assay showed the cell viability changes after *ADPGK* knockdown and AICAR addition (0.5 mmol/L) in LNCaP cells (*n* = 5). Data are presented as the mean ± SD. *P < 0.05, **P < 0.01, ***P < 0.001, ns non-significant. PCa prostate cancer, ADPGK ADP-dependent glucokinase, AMPK AMP-activated protein kinase, NC negative control, OE overexpression, ECAR extracellular acidification rate, 2-DG 2-deoxy-glucose, AICAR acadesine, OD optical density, SD standard deviation, a.u. artificial unit

AMPK activation could participate in glycolysis, the TCA cycle and cell cycle progression [29]. It was unclear whether ADPGK expression could alter the AMPK signaling pathway. Nevertheless, results indicated that ADPGK overexpression could increase the AMPK phosphorylation levels in 22Rv1 (Fig. 6a) and PC3 cells (Fig. 6b). In addition, siRNA-mediated *ADPGK* silencing decreased the AMPK phosphorylation levels in LNCaP cells (Fig. 6c). Furthermore, the ADPGK inhibitor decreased AMPK phosphorylation levels in 22Rv1 cells (Fig. 6d). To further validate whether the AMPK signaling pathway was regulated by ADPGK, an ECAR test and CCK-8 assay were performed in ADPGK OE cells after supplementation with the AMPK inhibitor Compound C. The glycolytic capacity (Fig. 6e) and cell viability (Fig. 6f) in ADPGK OE cells were decreased by Compound C. In addition, the glycolytic capacity (Fig. 6g) and cell viability (Fig. 6h) in *ADPGK* knockdown LNCaP cells were increased by the AMPK agonist AICAR. Collectively, these findings indicated that ADPGK promotes PCa glycolysis by activating the AMPK pathway.

### ADPGK binds with ALDOC to activate the AMPK pathway

**Fig. 7:**
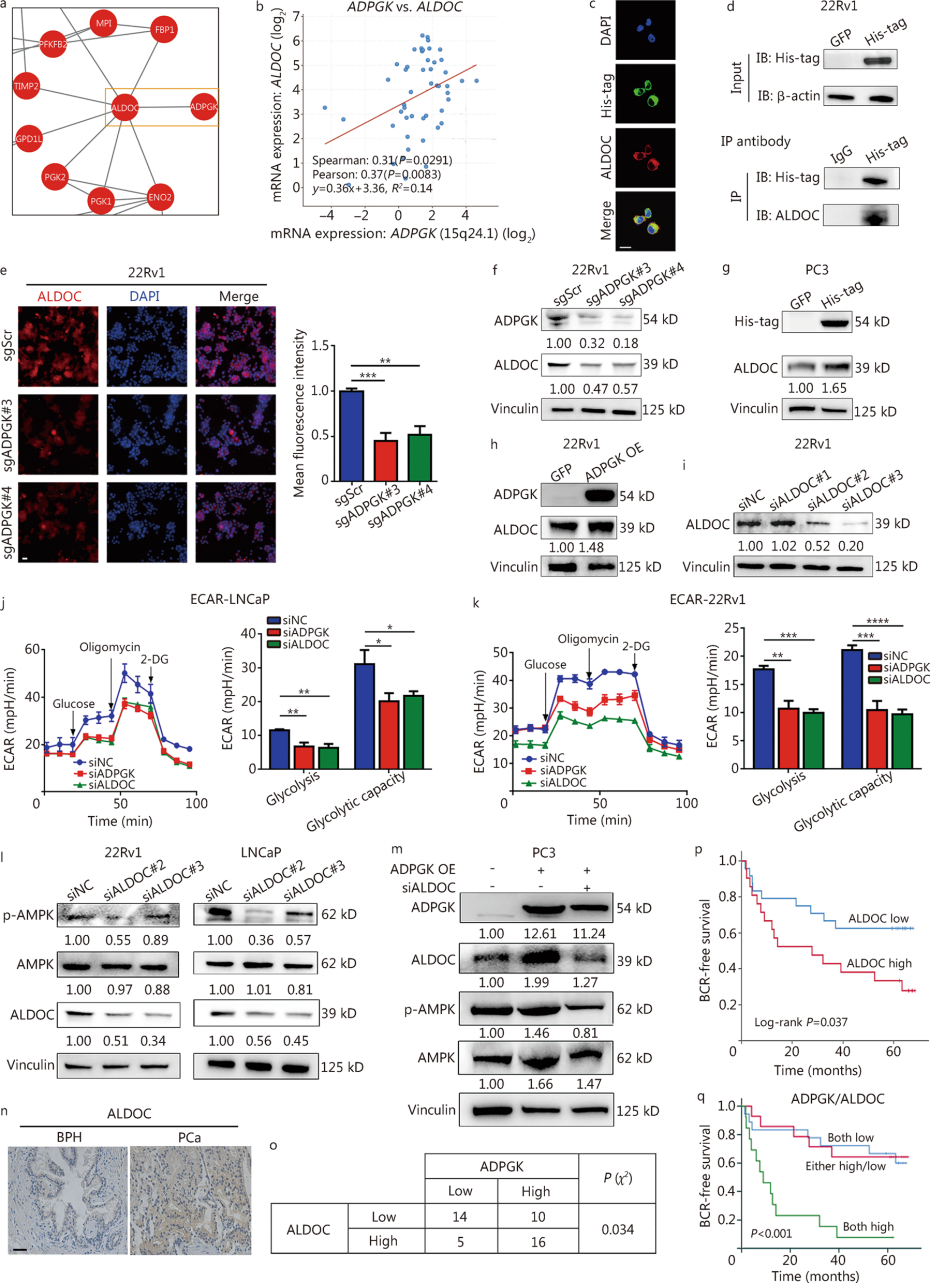
ADPGK binds with ALDOC, activating the AMPK pathway. **a** Protein–protein interaction network analysis from quantitative proteomics. **b** Correlation analysis between *ADPGK* and *ALDOC* expression from cBioPortal (data from Neuroendocrine Prostate Cancer Multi-Institute). **c** Immunofluorescence staining for His-tag (blue) and ALDOC (red) primary antibodies in PC3-ADPGK-His-tag cells. The mean colocalization Pearson’s correlation coefficient was 0.78 (*n* = 3). Scale bar = 20 μm. **d** Co-immunoprecipitation experiments showed the interactions between ADPGK and ALDOC (5 μg of His-tag and IgG IP antibody). **e** ALDOC expression in 22Rv1 cells after *ADPGK* knockdown, as detected by an immunofluorescence assay. The mean immunofluorescence intensity was evaluated by ImageJ software (*n* = 5). Scale bar = 20 μm. **f** Effect of *ADPGK* knockdown on ALDOC expression in 22Rv1 cells detected by Western blotting. Effect of ADPGK overexpression on ALDOC expression in PC3 (**g**) and 22Rv1 (**h**) cells detected by Western blotting.** i** siRNA-mediated *ALDOC* knockdown detected by Western blotting. ECAR test in LNCaP (**j**) and 22Rv1 (**k**) cells after *ADPGK* or *ALDOC* knockdown (*n* = 3). **l** Effect of *ALDOC* knockdown on ALDOC, AMPK and p-AMPK expression in 22Rv1 and LNCaP cells. **m** Effect of ADPGK overexpression and siALDOC#2 on ADPGK, ALDOC, AMPK and p-AMPK expression in PC3 cells.** n** ALDOC expression status in clinical samples assessed by immunohistochemistry (IHC). Scale bar = 50 μm. **o** Representative IHC quantification of ADPGK and ALDOC expression status in PCa tissues (*χ*^2^ test). **p** Kaplan‒Meier curves showing the association of ALDOC expression with biochemical recurrence (BCR) in 45 patients. **q** Kaplan‒Meier curves showing survival outcomes among PCa patients stratified into three groups: ADPGK_high_/ALDOC_high_ group (*n* = 16), ADPGK_low_/ALDOC_high_ + ADPGK_high_/ALDOC_low_ group (*n* = 17), and ADPGK_low_/ALDOC_low_ group (*n* = 12). Data are presented as the mean ± SD. *P < 0.05, **P < 0.01, ***P < 0.001, ****P < 0.0001. PCa prostate cancer, BPH benign prostate hyperplasia, ADPGK ADP-dependent glucokinase, ALDOC aldolase C, AMPK AMP-activated protein kinase, NC negative control, OE overexpression, ECAR extracellular acidification rate, 2-DG 2-deoxy-glucose, SD standard deviation

To illustrate the underlying mechanisms of AMPK phosphorylation/activation by ADPGK, a protein–protein interaction network analysis was conducted using data from the quantitative proteomics analysis and found that ADPGK might interact with ALDOC to regulate glycolysis-related genes (Fig. 7a). Interestingly, previous studies suggested that the ALDOC-AMPK pathway could regulate cell glycolysis [30, 31]. Therefore, we hypothesized that ADPGK might interact with ALDOC to regulate the AMPK signaling pathway. Data from the cBioPortal database (neuroendocrine PCa [32]) revealed a positive correlation between *ADPGK* mRNA expression and *ALDOC* mRNA expression (Fig. 7b). The ADPGK was overexpressed fused with a 6 × His-tag in PC3 cells, and immunofluorescence assays revealed significant colocalization of ADPGK and ALDOC in PC3 cells (Fig. 7c, the mean Pearson’s correlation coefficient was 0.78). Co-IP experiments further revealed that ADPGK could directly bind with ALDOC proteins (Fig. 7d). In addition, subsequent immunofluorescence (Fig. 7e) and Western blotting (Fig. 7f) assays demonstrated that ALDOC expression was decreased after *ADPGK* knockdown. In addition, ADPGK OE also increased ALDOC protein expression in PC3 (Fig. 7g) and 22Rv1 cells (Fig. 7h). *ADPGK* or *ALDOC* knockdown (Fig. 7i) could also inhibit glycolysis and glycolytic capacity in LNCaP (Fig. 7j) and 22Rv1 (Fig. 7k) cells. *ALDOC* silencing decreased the AMPK phosphorylation levels (Fig. 7l), and the increased AMPK phosphorylation levels resulting from ADPGK OE could be inhibited after *ALDOC* knockdown (Fig. 7m).

**Fig. 8:**
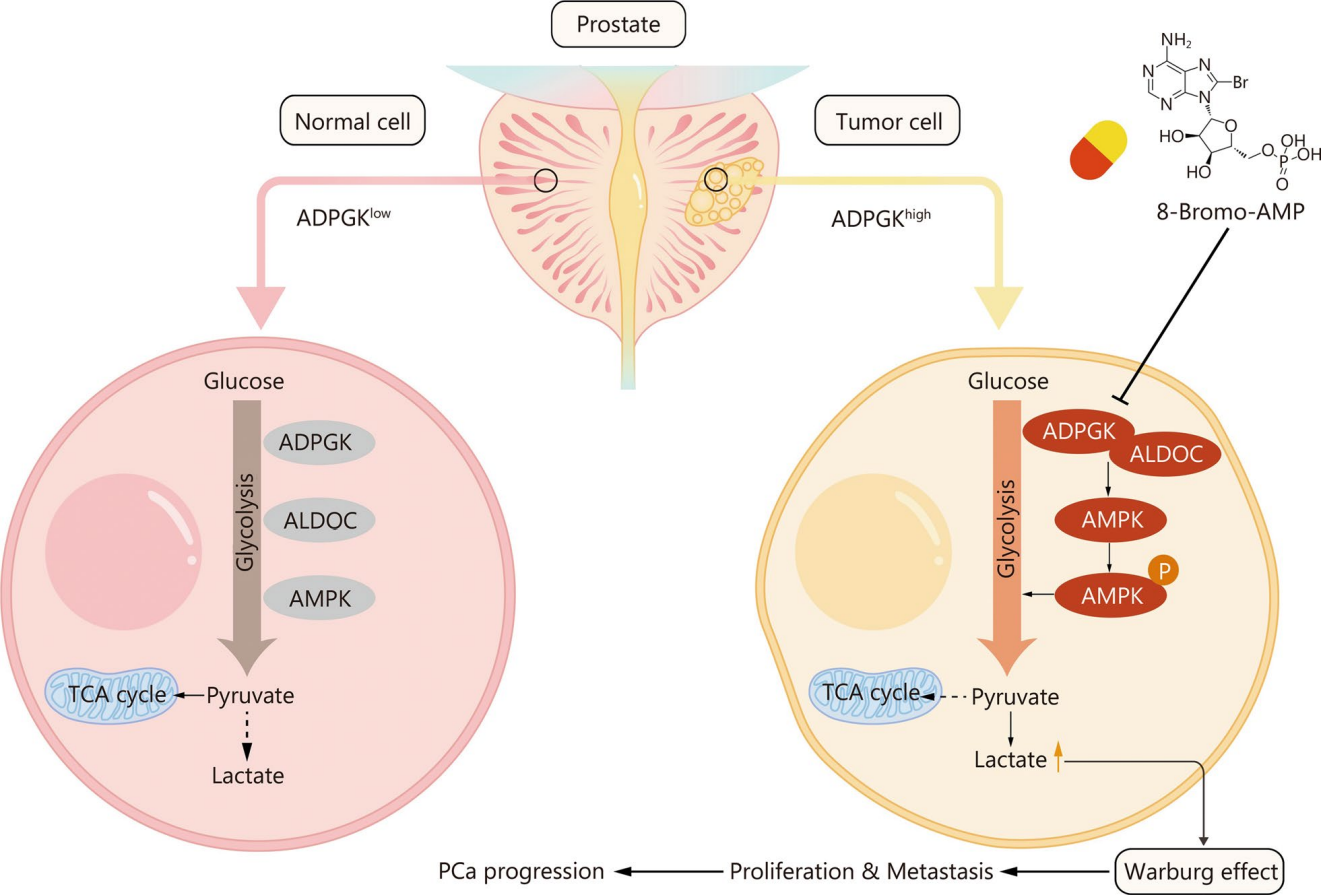
Schematic diagram shows that ADPGK regulates metabolic fitness in PCa progression. ADPGK overexpression significantly promotes the process of glycolysis, and the overproduction of lactate induced Warburg effect further orchestrates the PCa progression. PCa prostate cancer, ADPGK ADP-dependent glucokinase, ALDOC aldolase C, AMPK AMP-activated protein kinase, TCA tricarboxylic acid

PCa patient data from West China Hospital indicated that ALDOC was highly expressed in PCa tissues compared with BPH tissues (Fig. 7n). After analyzing ADPGK and ALDOC expression in PCa tissues, it appeared that they were also correlated with each other (*P* = 0.034, Fig. 7o). High ALDOC expression was associated with a short BCR time (Fig. 7p). The combination of ADPGK and ALDOC expression revealed that patients with high ADPGK and ALDOC expression had a significantly shorter BCR time than those with low expression (Fig. 7q). Taken together, these results indicated that ADPGK interacts with ALDOC to activate AMPK phosphorylation and therefore promote PCa progression (Fig. 8).

## Discussion

Current treatment approaches for patients with advanced PCa, especially CRPC, are diverse and include endocrine therapy (abiraterone [33] and enzalutamide [33]), chemotherapy [34], radiation therapy [35], targeted therapy [36] and immune therapy [37], but their efficacies are still unsatisfactory. Thus, strategies for increasing treatment efficacy and survival time are needed.

Recent studies have suggested that cell metabolism plays a pivotal role in tumor progression; thus, targeting cancer metabolism to kill cancer cells might be an effective approach [38–40]. The conversion of glucose to G6P is the central biochemical event of cell metabolism. In this study, among all the HKs, *ADPGK* was the only gene that both upregulated and predicted OS in PRAD through bioinformatic analysis. Further results from PCa clinical tissue samples proved that ADPGK was significantly highly expressed in PCa tissues, and the higher the expression of ADPGK was, the worse the prognosis of PCa was. ADPGK modulated PCa cell proliferation and migration. The team further investigated the underlying mechanism and found that ADPGK interacts with ALDOC to regulate PCa metabolic fitness by activating AMPK signaling. To our knowledge, this was the first study to investigate the mechanism of ADPGK in the progression of PCa. These data together provide new insights into the mechanisms of PCa growth and metastasis and suggest that ADPGK might be a potential therapeutic target for PCa.

ADPGK is an enzyme involved in the glycolytic pathway, and the results of this study showed that the effect of ADPGK on the progression of PCa was regulated by glycolysis. The relationship between glycolysis and tumorigenesis has been confirmed by numerous studies. The use of ATP between normal cells and tumor cells is different. Normal cells generally produce energy through glycolysis only when they are in a hypoxic state, while tumor cells rely on glycolysis for energy supply even when oxygen is abundant, which is known as the Warburg effect [41]. High glucose uptake, aerobic glycolysis and high levels of lactate production are the main manifestations of the Warburg effect [42, 43], which were validated by metabolomics findings of high lactate production and by ECAR test findings of high glycolytic capacity after ADPGK overexpression and low glycolytic capacity after *ADPGK* knockdown.

In addition, a study has shown that a variety of intermediates during glycolysis can be used by tumor cells to synthesize essential proteins and lipids, which will inevitably lead to increased activity of enzymes related to the glycolytic pathway and reduced intermediates [44]. This study proposed that ADPGK expression can significantly increase the levels of glycolysis-related enzymes and decrease the levels of glycolytic intermediate products such as phosphoenolpyruvate, G6P, 3-phosphoglyceraldehyde and 2,3-diphosphoglycerate compared with those of the control group. Most crucially, through performing protein–protein interaction analysis, we found that ADPGK has a direct interaction with ALDOC, which was also validated in subsequent Co-IP assay. A previous study revealed that aldolases can sense glucose absence, contributing to AMPK signaling activation [31], and Fan et al. [30] found that MUC16c binding with ALDOC promotes the phosphorylation of AMPK in gallbladder carcinoma (GBC). Consistent with these findings, this study demonstrated that ADPGK might regulate the ALDOC-AMPK pathway to promote PCa glycolysis and progression. Moreover, our team also confirmed that ALDOC is highly expressed in PCa tissues and that patients with high ALDOC expression had a short BCR time. We hope this work provides a new mechanism of PCa cell metabolism and might be validated in other tumors.

The role of ADPGK in tumorigenesis and development is currently unclear, and relevant studies are sparse. In 2012, Richter et al. [20] studied the expression and role of ADPGK in tumor cells, and their results confirmed that ADPGK expression was not affected by hypoxic stimulation or hypoxia inducible factor-1, and overexpression of ADPGK did not increase the glycolytic levels in H460 and HCT116 cells. When the mRNA of *ADPGK* in H460 cells was knocked down, glycolysis was not affected, but the colony formation of H460 cells was significantly reduced. In 2019, Imle et al. [21] demonstrated that *ADPGK* knockout could promote apoptosis and increase endoplasmic reticulum stress in Jurkat T cells. After *ADPGK* knockout, Jurkat T cells exhibited severe energy metabolic disorder, impeding the Warburg effect. Further verification was also carried out in zebrafish models, where the deletion of the *ADPGK* gene in zebrafish embryos led to increased apoptosis, resulting in a shortened body axis and dorsalization and further dysregulation of glucose metabolism in zebrafish. Due to the high heterogeneity across different tumors, it is unclear whether these two inconsistent results apply to PCa and whether ADPGK can regulate glycolysis in PCa cells. This study provides the first evidence that ADPGK can accelerate PCa cell glycolysis via ALDOC-AMPK pathway activation.

## Conclusions

Our study is the first to reveal that ADPGK is a driving factor in the progression of PCa and contributes to the poor prognosis of PCa patients. ADPGK might accelerate PCa glycolysis and progression via ALDOC-AMPK signaling activation. These findings indicate that ADPGK can be used as a target and marker for PCa, providing a new opportunity for effective treatment and prognosis evaluation.

### Supplementary Information

**Additional file 1**. **Table S1** Primary antibodies used in this study. **Table S2** siRNA sequences. **Table S3** sgRNAs sequences. **Table S4** Primers used in this study. Table S5 Univariate Cox regression analysis. **Fig. S1** Function and expression of genes involved in the oxidative phosphorylation of glucose and their associations with clinical outcomes. **Fig. S2** Cell type enrichment and prognosis of PRAD subgroups based on ADPGK expression. **Fig. S3** Association between ADPGK and PCa immune status. **Fig. S4** qPCR results showed the stable overexpression of ADPGK in PCa cells after lentivirus transfection. **Fig. S5** The impact of ADPGK knockdown on LNCaP cell viability was assessed by CCK-8 assay. **Fig. S6** Hematoxylin and eosin (HE) staining of lung tissues of mice bearing PC3 cells.

## Data Availability

The data and materials used in the current study are available from the corresponding author upon reasonable request.

## Abbreviations

ADPGK: ADP-dependent glucokinase
ADT: Androgen deprivation therapy
ALDOC: Aldolase C
AMPK: AMP-activated protein kinase
ATP: Adenosine triphosphate
AUC: Area under curve
BCR: Biochemical recurrence
BPH: Benign prostate hyperplasia
Co-IP: Co-immunoprecipitation
CRPC: Castration-resistant prostate cancer
ECAR: Extracellular acidification rate
EdU: 5-Ethynyl-2'-deoxyuridine
EPE: Extraprostatic extension
GBC: Gallbladder carcinoma
GS: Gleason score
HE: Haematoxylin and eosin
HRC: Heat release capacity
IHC: Immunohistochemistry
KEGG: Kyoto Encyclopedia of Genes and Genomes
OCR: Oxygen consumption rate
OS: Overall survival
PBS: Phosphate buffer saline
PCa: Prostate cancer
PNI: Perineural invasion
PSA: Prostate specific antigen
PSM: Positive surgical margin
qPCR: Quantitative polymerase chain reaction
RARP: Robot-assisted laparoscopic radical prostatectomy
READ: Rectum adenocarcinoma
ROC: Receiver operating characteristic
siRNA: Small interfering RNA
STAD: Stomach adenocarcinoma
SVI: Seminal vesicle invasion
TCA: Tricarboxylic acid
TCGA: The Cancer Genome Atlas
UCEC: Uterine corpus endometrial carcinoma

## References

[1] Siegel RL, Miller KD, Wagle NS, Jemal A (2023). Cancer statistics, 2023. CA Cancer J Clin.

[2] Zi H, He SH, Leng XY, Xu XF, Huang Q, Weng H (2021). Global, regional, and national burden of kidney, bladder, and prostate cancers and their attributable risk factors, 1990–2019. Mil Med Res.

[3] Mottet N, van den Bergh RCN, Briers E, Van den Broeck T, Cumberbatch MG, De Santis M (2021). EAU-EANM-ESTRO-ESUR-SIOG Guidelines on Prostate Cancer-2020 Update Part 1: screening, diagnosis, and local treatment with curative intent. Eur Urol..

[4] Rebello RJ, Oing C, Knudsen KE, Loeb S, Johnson DC, Reiter RE (2021). Prostate cancer. Nat Rev Dis Primers.

[5] Teo MY, Rathkopf DE, Kantoff P (2019). Treatment of advanced prostate cancer. Annu Rev Med.

[6] Cai M, Song XL, Li XA, Chen M, Guo J, Yang DH (2023). Current therapy and drug resistance in metastatic castration-resistant prostate cancer. Drug Resist Updat.

[7] Zhu J, Thompson CB (2019). Metabolic regulation of cell growth and proliferation. Nat Rev Mol Cell Biol.

[8] Paul S, Ghosh S, Kumar S (2022). Tumor glycolysis, an essential sweet tooth of tumor cells. Semin Cancer Biol.

[9] Koppenol WH, Bounds PL, Dang CV (2011). Otto Warburg's contributions to current concepts of cancer metabolism. Nat Rev Cancer.

[10] Semenza GL, Artemov D, Bedi A, Bhujwalla Z, Chiles K, Feldser D (2001). The metabolism of tumours: 70 years later. Novartis Found Symp..

[11] Wilson JE (2003). Isozymes of mammalian hexokinase: structure, subcellular localization and metabolic function. J Exp Biol.

[12] Xu S, Herschman HR (2019). A tumor agnostic therapeutic strategy for hexokinase 1-null/hexokinase 2-positive cancers. Cancer Res.

[13] Jiao L, Zhang HL, Li DD, Yang KL, Tang J, Li X (2018). Regulation of glycolytic metabolism by autophagy in liver cancer involves selective autophagic degradation of HK2 (hexokinase 2). Autophagy.

[14] Liu Y, Murray-Stewart T, Casero RA, Kagiampakis I, Jin L, Zhang J (2017). Targeting hexokinase 2 inhibition promotes radiosensitization in HPV16 E7-induced cervical cancer and suppresses tumor growth. Int J Oncol.

[15] Pudova EA, Kudryavtseva AV, Fedorova MS, Zaretsky AR, Shcherbo DS, Lukyanova EN (2018). HK3 overexpression associated with epithelial-mesenchymal transition in colorectal cancer. BMC Genomics.

[16] Wang L, Wang J, Xiong H, Wu F, Lan T, Zhang Y (2016). Co-targeting hexokinase 2-mediated Warburg effect and ULK1-dependent autophagy suppresses tumor growth of PTEN- and TP53-deficiency-driven castration-resistant prostate cancer. EBioMedicine.

[17] Hruz T, Laule O, Szabo G, Wessendorp F, Bleuler S, Oertle L (2008). Genevestigator v3: a reference expression database for the meta-analysis of transcriptomes. Adv Bioinform.

[18] Ronimus RS, Morgan HW (2004). Cloning and biochemical characterization of a novel mouse ADP-dependent glucokinase. Biochem Biophys Res Commun.

[19] Kaminski MM, Sauer SW, Kaminski M, Opp S, Ruppert T, Grigaravicius P (2012). T cell activation is driven by an ADP-dependent glucokinase linking enhanced glycolysis with mitochondrial reactive oxygen species generation. Cell Rep.

[20] Richter S, Richter JP, Mehta SY, Gribble AM, Sutherland-Smith AJ, Stowell KM (2012). Expression and role in glycolysis of human ADP-dependent glucokinase. Mol Cell Biochem.

[21] Imle R, Wang BT, Stutzenberger N, Birkenhagen J, Tandon A, Carl M (2019). ADP-dependent glucokinase regulates energy metabolism via ER-localized glucose sensing. Sci Rep.

[22] Uhlén M, Fagerberg L, Hallström BM, Lindskog C, Oksvold P, Mardinoglu A (2015). Proteomics. Tissue-based map of the human proteome. Science.

[23] Yin C, Zhu B, Zhang T, Liu T, Chen S, Liu Y (2019). Pharmacological targeting of STK19 inhibits oncogenic NRAS-driven melanomagenesis. Cell.

[24] Xu H, Zhang J, Zheng X, Tan P, Xiong X, Yi X (2022). SR9009 inhibits lethal prostate cancer subtype 1 by regulating the LXRα/FOXM1 pathway independently of REV-ERBs. Cell Death Dis.

[25] Andersen C, Kotowska D, Tortzen CG, Kristiansen K, Nielsen J, Petersen RK (2014). 2-(2-Bromophenyl)-formononetin and 2-heptyl-formononetin are PPARγ partial agonists and reduce lipid accumulation in 3T3-L1 adipocytes. Bioorg Med Chem.

[26] Yu PB, Hong CC, Sachidanandan C, Babitt JL, Deng DY, Hoyng SA (2008). Dorsomorphin inhibits BMP signals required for embryogenesis and iron metabolism. Nat Chem Biol.

[27] Grudnik P, Kamiński MM, Rembacz KP, Kuśka K, Madej M, Potempa J (2018). Structural basis for ADP-dependent glucokinase inhibition by 8-bromo-substituted adenosine nucleotide. J Biol Chem.

[28] Yang J, Das BC, Aljitawi O, Kumar A, Das S, Van Veldhuizen P (2022). Magmas inhibition in prostate cancer: a novel target for treatment-resistant disease. Cancers.

[29] Hsu CC, Peng D, Cai Z, Lin HK (2022). AMPK signaling and its targeting in cancer progression and treatment. Semin Cancer Biol.

[30] Fan K, Wang J, Sun W, Shen S, Ni X, Gong Z (2020). MUC16 C-terminal binding with ALDOC disrupts the ability of ALDOC to sense glucose and promotes gallbladder carcinoma growth. Exp Cell Res.

[31] Zhang CS, Hawley SA, Zong Y, Li M, Wang Z, Gray A (2017). Fructose-1,6-bisphosphate and aldolase mediate glucose sensing by AMPK. Nature.

[32] Beltran H, Prandi D, Mosquera JM, Benelli M, Puca L, Cyrta J (2016). Divergent clonal evolution of castration-resistant neuroendocrine prostate cancer. Nat Med.

[33] James ND, de Bono JS, Spears MR, Clarke NW, Mason MD, Dearnaley DP (2017). Abiraterone for prostate cancer not previously treated with hormone therapy. N Engl J Med.

[34] Oudard S, Fizazi K, Sengeløv L, Daugaard G, Saad F, Hansen S (2017). Cabazitaxel versus docetaxel as first-line therapy for patients with metastatic castration-resistant prostate cancer: a randomized phase III trial-FIRSTANA. J Clin Oncol.

[35] Parker C, Nilsson S, Heinrich D, Helle SI, O'Sullivan JM, Fosså SD (2013). Alpha emitter radium-223 and survival in metastatic prostate cancer. N Engl J Med.

[36] Mateo J, Carreira S, Sandhu S, Miranda S, Mossop H, Perez-Lopez R (2015). DNA-repair defects and olaparib in metastatic prostate cancer. N Engl J Med.

[37] Abida W, Cheng ML, Armenia J, Middha S, Autio KA, Vargas HA (2019). Analysis of the prevalence of microsatellite instability in prostate cancer and response to immune checkpoint blockade. JAMA Oncol.

[38] Altman BJ, Stine ZE, Dang CV (2016). From Krebs to clinic: glutamine metabolism to cancer therapy. Nat Rev Cancer.

[39] Pavlova NN, Thompson CB (2016). The emerging hallmarks of cancer metabolism. Cell Metab.

[40] Yuan S, Fang C, Leng WD, Wu L, Li BH, Wang XH (2021). Oral microbiota in the oral-genitourinary axis: identifying periodontitis as a potential risk of genitourinary cancers. Mil Med Res.

[41] Racker E (1972). Bioenergetics and the problem of tumor growth. Am Sci.

[42] Cairns RA, Harris IS, Mak TW (2011). Regulation of cancer cell metabolism. Nat Rev Cancer.

[43] Levine AJ, Puzio-Kuter AM (2010). The control of the metabolic switch in cancers by oncogenes and tumor suppressor genes. Science.

[44] Teslaa T, Teitell MA (2015). Pluripotent stem cell energy metabolism: an update. Embo j.

