# Supplementary material for: ADP-dependent glucokinase controls metabolic fitness in prostate cancer progression
Source: Mil Med Res. 2023 Dec 12;10:64. doi: 10.1186/s40779-023-00500-9 (PMC10714548; doi:10.1186/s40779-023-00500-9)
Supplement: Supplementary file 1 — Additional file 1. Table S1 Primary antibodies used in this study. Table S2 siRNA sequences. Table S3 sgRNAs sequences. Table S4 Primers used in this study. Table S5 Univariate Cox regression analysis. Fig. S1 Function and expression of genes involved in the oxidative phosphorylation of glucose and their associations with clinical outcomes. Fig. S2 Cell type enrichment and prognosis of PRAD subgroups based on ADPGK expression. Fig. S3 Association between ADPGK and PCa immune status. Fig. S4 qPCR results showed the stable overexpression of ADPGK in PCa cells after lentivirus transfection. Fig. S5 The impact of ADPGK knockdown on LNCaP cell viability was assessed by CCK-8 assay. Fig. S6 Hematoxylin and eosin (HE) staining of lung tissues of mice bearing PC3 cells. [file 40779_2023_500_MOESM1_ESM.pdf]

Table S1 Primary antibodies used in this study

Antigens	Application	Host	Manufacturer	Cat. number
β-actin	1:5000	Mouse	Zen BioScience	250132
ADPGK	1:500 – 1:3000	Rabbit	Abcam	ab228633
CCND1	1:500 – 1:2000	Rabbit	Zen BioScience	382442
β-tubulin	1:500 – 1:2000	Rabbit	Zen BioScience	380628
ALDOC	1:500 – 1:2000	Rabbit	Zen BioScience	611464
GAPDH	1:5000 – 1:20,000	Mouse	Zen BioScience	200306-7E4
AMPK α2	1:1000	Rabbit	Zen BioScience	R23464
p-AMPK α2	1:500 – 1:2000	Rabbit	Zen BioScience	381164
His-tag	1:5000	Rabbit	Abcam	ab213204
ALDOC	1:5000	Mouse	Proteintech	66120-1-Ig

ADPGK ADP-dependent glucokinase, *CCND1* cyclin D1, *ALDOC* aldolase C, *AMPK* AMP-activated protein kinase

Table S2 siRNA sequences

siRNA number	Sequence (5' – 3')
siADPGK #1	GGAACGGTGTTCCTGATGT
siADPGK #2	TGAGCAGCATTGTCCATCA
siADPGK #3	GCAGCCAACTCAGATTTAA
siALDOC #1	GCAGCACAGTCACTCTACA
siALDOC #2	CCTCAAACGTTGTCAGTAT
siALDOC #3	GAACGCTGTGCCCAATACA

ADPGK ADP-dependent glucokinase, *ALDOC* aldolase C

Table S3 sgRNAs sequences

Gene name	Forward primer (5' – 3')	Reverse primer (5' – 3')
sgADPGK#2	caccgGTCAATGCATGTGTTGATG	aaacCATCAACACATGCATTGACc
sgADPGK#3	caccgTCTCTCACGACCTCTCCAA	aaacTTGGAGAGGTCGTGAGAGAc
sgADPGK#4	caccgTGCTTGATACTCTAAAATG	aaacCATTTTAGAGTATCAAGCAc

ADPGK ADP-dependent glucokinase

Table S4 Primers used in this study

Gene name	Forward primer (5' – 3')	Reverse primer (5' – 3')
ADPGK	CGTGGCAGTGGGAGTCAAT	TGAATGCAGAATGCTGTGATCT
ACTB	CATGTACGTTGCTATCCAGGC	CTCCTTAATGTCACGCACGAT

ADPGK ADP-dependent glucokinase, *ACTB* β-actin

Table S5 Univariate Cox regression analysis

**Variables**	**BCR**	
	***HR* (95%CI)**	***P***
Age (≥ 65 years vs. < 65 years)	1.155 (0.361 – 3.692)	0.808
Baseline PSA (≥ 20 ng/ml vs. < 20 ng/ml)	2.854 (0.893 – 9.121)	0.077
BMI (≥ 25 kg/m^2 vs. < 25 kg/m^2)	0.976 (0.326 – 2.925)	0.966
Neoadjuvant ADT (yes vs. no)	5.070 (1.717 – 14.975)	0.003
ADPGK expression (high vs. low)	3.785 (1.048 – 13.673)	0.042
pT stage (≥ pT3 vs. < pT3)	2.591 (0.811 – 8.281)	0.108
GS (≥ 8 vs. < 8)	3.110 (1.039 – 9.310)	0.043
EPE (+ vs. -)	2.591 (0.811 – 8.281)	0.108
SVI (+ vs. -)	4.284 (1.475 – 12.446)	0.007
PNI (+ vs. -)	3.168 (0.707 – 14.201)	0.132
PSM (+ vs. -)	2.310 (0.517 – 10.327)	0.273
ADT (yes vs. no)	6.202 (1.385 – 27.764)	0.017
Adjuvant radiotherapy (yes vs. no)	1.910 (0.668 – 5.465)	0.228
Post RARP 3-month PSA		0.093
< 0.003 ng/ml	Ref.	
0.003 – 0.200 ng/ml	0.823 (0.206 – 3.293)	0.783
> 0.200 ng/ml	2.989 (0.704 – 12.680)	0.138

BCR biochemical recurrence, *HR* hazard ratio, *CI* confidence interval, *PSA* prostate specific antigen, *BMI* body mass index, *ADT* androgen deprivation therapy, *pT* pathologic T, *GS* Gleason score, *EPE* extraprostatic extension, *SVI* seminal vesicle invasion, *PNI* perineural invasion, *PSM* positive surgical margin, *RARP* robot-assisted laparoscopic radical prostatectomy, *ADPGK* ADP-dependent glucokinase, *Ref.* reference

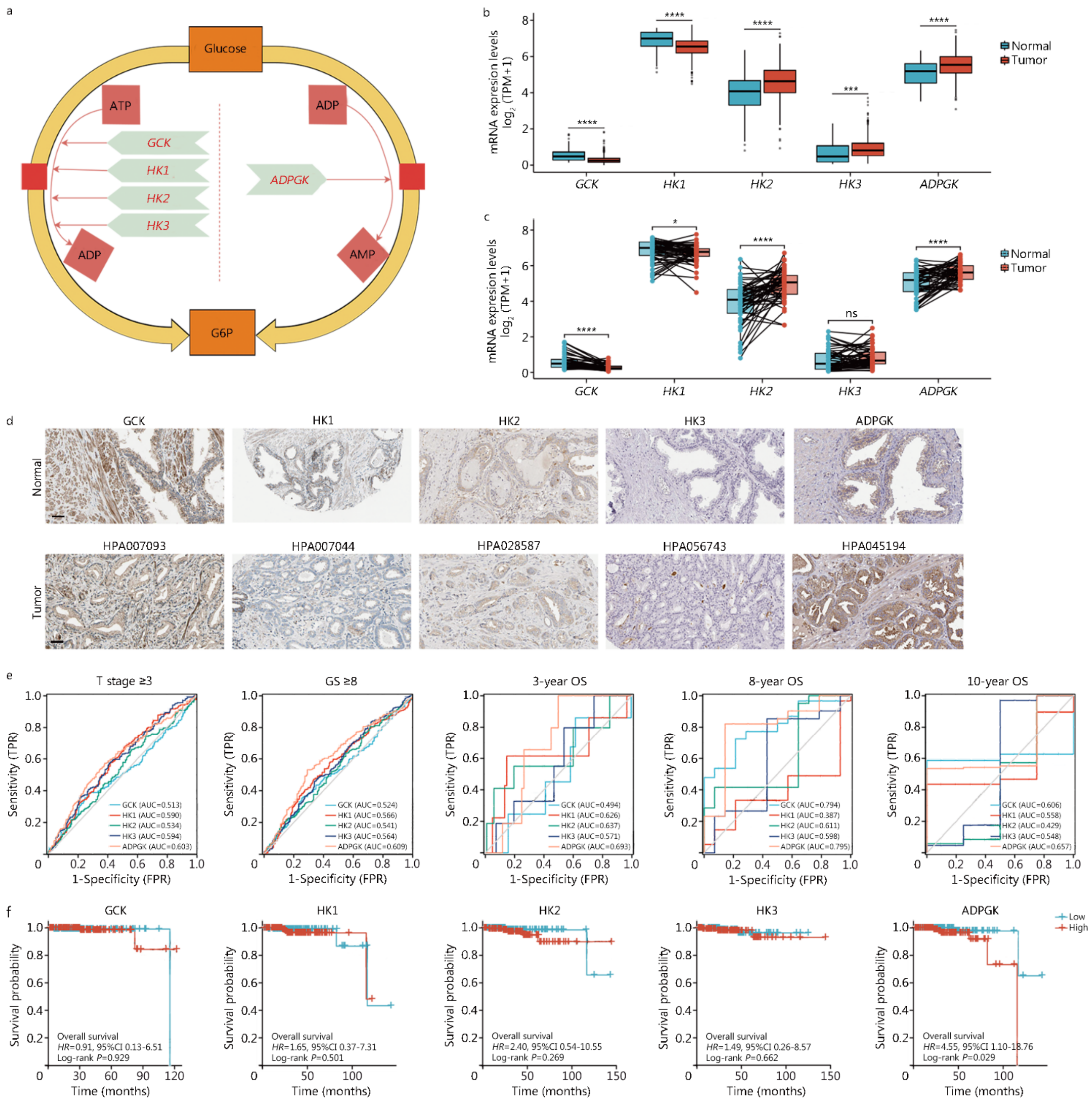

Fig. S1 Function and expression of genes involved in the oxidative phosphorylation of glucose and their associations with clinical outcomes. **a** Five genes including *GCK*, *HK1*, *HK2*, *HK3* and *ADPGK* were involved in the conversion of glucose to glucose-6-phosphate (G6P). mRNA expressions of *GCK*, *HK1*, *HK2*, *HK3* and *ADPGK* in TCGA-PRAD samples compared with normal tissues (**b**, n = 553) or paired normal tissues (**c**, n = 104) were showed. **d** Immunohistochemistry results from the Human Protein Atlas showed the protein expression of GCK, HK1, HK2, HK3 and ADPGK in normal and PCa tissues. Scale bar = 50 μm. **e** Sensitivities and specificities of GCK, HK1, HK2, HK3 and ADPGK in predicting high T stage (≥ 3), high Gleason score (GS; ≥ 8), and 3-, 8- and 10-year OS were showed in ROC curves (n = 496). **f** Survival curves of GCK, HK1, HK2, HK3 and ADPGK in predicting PCa OS were exhibited (n = 496). $^{*}P < 0.05$, $^{***}P < 0.001$, $^{****}P < 0.0001$. ns non-significant, TCGA The Cancer Genome

Atlas, PRAD prostate adenocarcinoma, ATP adenosine triphosphate, ADP adenosine diphosphate, AMP adenosine monophosphate, GCK glucokinase, HK hexokinase, ADPGK ADP-dependent glucokinase, OS overall survival, *HR* hazard ratio, TPR true positive rate, FPR false positive rate

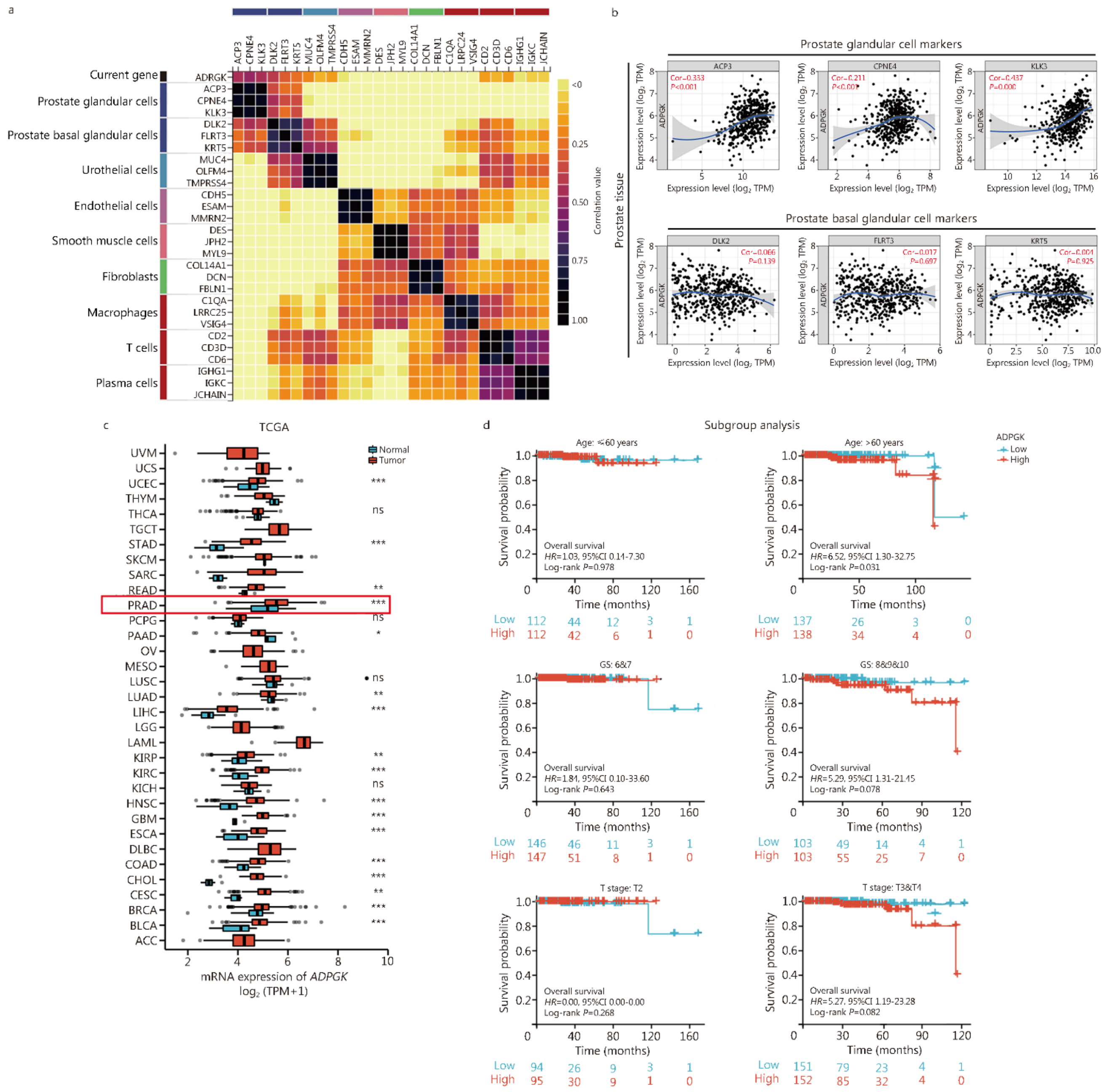

Fig. S2 Cell type enrichment and prognosis of PRAD subgroups based on ADPGK expression. **a** ADPGK was significantly correlated with prostate glandular cell markers (ACP3, CPNE4, KLK3, DLK2, FLRT3, and KRT5). **b** In PCa, ADPGK was significantly positively correlated with ACP3 (also known as ACPP), CPNE4, and KLK3. **c** *ADPGK* mRNA expression differences were analyzed in pan-cancer analysis. **d** Subgroup analyses were conducted according to PRAD patients' age, Gleason score (GS) and T stage. $^{*}P < 0.05$, $^{**}P < 0.01$. $^{***}P < 0.001$. ns non-significant. ADPGK ADP-dependent glucokinase, TCGA The Cancer Genome Atlas, PRAD prostate adenocarcinoma, *HR* hazard ratio, ACP3 prostatic acid phosphatase, CPNE4 copine 4, KLK3 kallikrein-3, DLK2 delta-like 2, FLRT3 fibronectin leucine rich transmembrane protein 3, KRT5 keratin 5, MUC4 mucin 4, OLFM4 olfactomedin 4, TMPRSS4 transmembrane protease serine 4, CDH5 cadherin 5, ESAM endothelial cell-specific adhesion molecule, MMRN2 multimerin-2, DES desmin, JPH2 junctophilin-2, MYL9 myosin light chain 9,

COL14A1 collagen, type XIV, alpha 1, DCN decorin, FBLN1 fibulin 1, C1QA complement component 1, q subcomponent, A chain, LRRC25 leucine rich repeat containing 25, VSIG4 V-set and immunoglobulin domain containing 4, IGHG1 immunoglobulin heavy constant gamma 1, IGKC immunoglobulin kappa constant, JCHAIN joining chain of multimeric IgA and IgM, UVM uveal melanoma, UCS uterine carcinosarcoma, UCEC uterine corpus endometrial carcinoma, THYM thymoma, THCA thyroid carcinoma, TGCT testicular germ cell tumors, STAD stomach adenocarcinoma, SKCM skin cutaneous melanoma, SARC sarcoma, READ rectum adenocarcinoma, PCPG pheochromocytoma and paraganglioma, PAAD pancreatic adenocarcinoma, OV ovarian serous cystadenocarcinoma, MESO mesothelioma, LUSC lung squamous cell carcinoma, LUAD lung adenocarcinoma, LIHC liver hepatocellular carcinoma, LGG brain lower grade glioma, LAML acute myeloid leukemia, KIRP kidney renal papillary cell carcinoma, KIRC kidney renal clear cell carcinoma, KICH kidney chromophobe, HNSC head and neck squamous cell carcinoma, GBM glioblastoma multiforme, ESCA esophageal carcinoma, DLBC lymphoid neoplasm diffuse large B-cell lymphoma, COAD colon adenocarcinoma, CHOL cholangiocarcinoma, CESC cervical squamous cell carcinoma and endocervical adenocarcinoma, BRCA breast invasive carcinoma, BLCA bladder urothelial carcinoma, ACC adrenocortical carcinoma

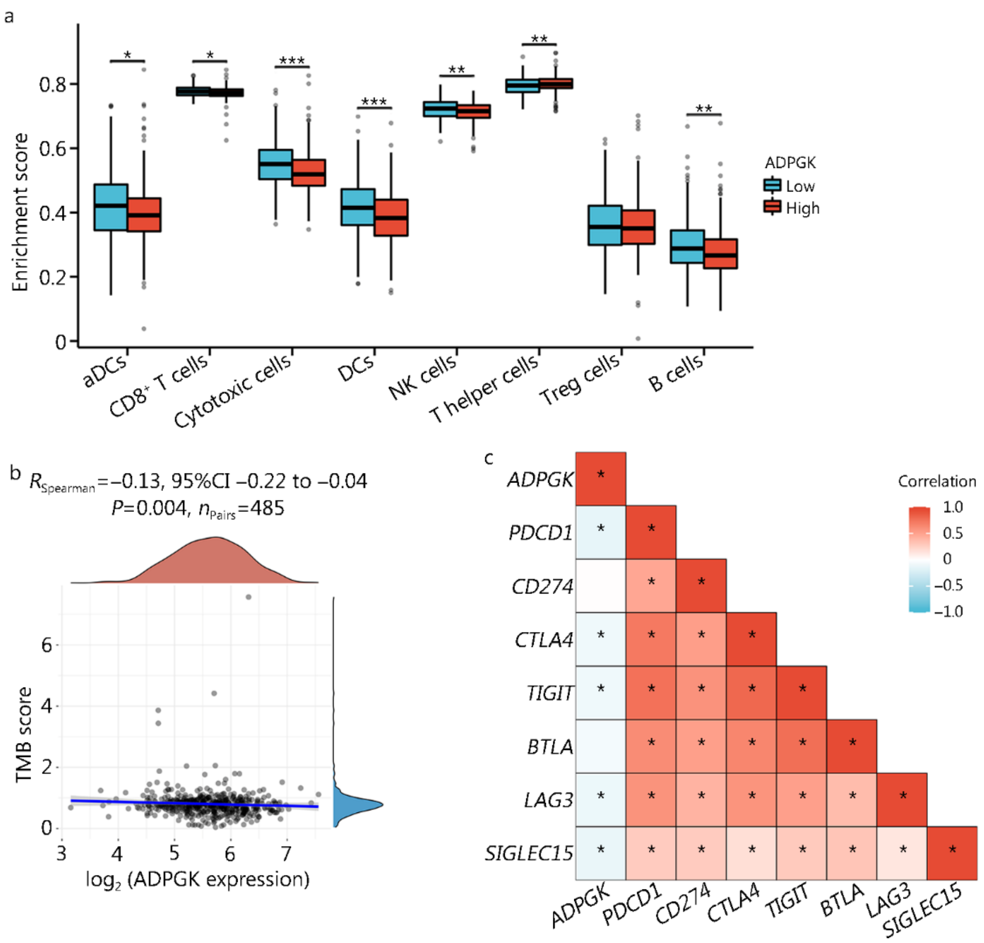

Fig. S3 Association between ADPGK and PCa immune status. **a**. Immune cell infiltration comparison between ADPGK high and low groups in PCa were analyzed with ssGSEA algorithm using R package "GSVA". **b** Correlation analysis between ADPGK expression and TMB in PCa was performed using Spearman's method. **c** Correlation analysis between ADPGK and immune checkpoint genes expression was performed using Spearman's method with R package "ggplot2". $^{*}P < 0.05$, $^{**}P < 0.01$, $^{***}P < 0.001$. ADPGK ADP-dependent glucokinase, PCa prostate cancer, DC dendritic cell, NK natural killer, Treg regulatory T, GSEA Gene Set Enrichment Analysis, TMB tumor mutational burden

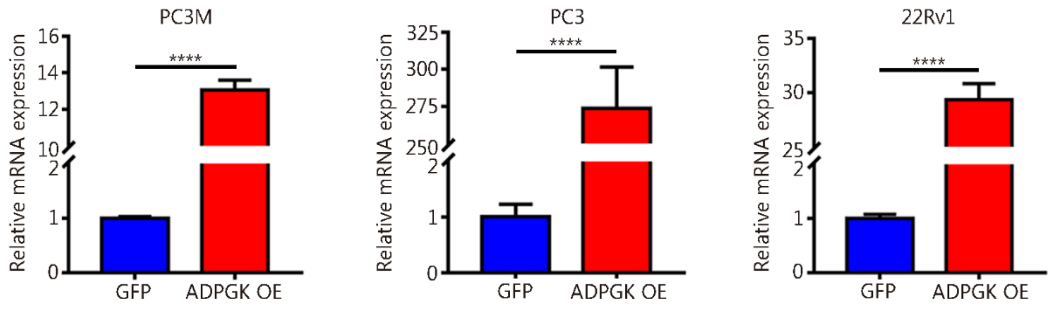

Fig. S4 qPCR results showed the stable overexpression of ADPGK in PCa cell lines after lentivirus transfection. Data were presented as the mean ± SD (n = 3). $^{***}P < 0.001$, $^{****}P < 0.0001$. qPCR quantitative polymerase chain reaction, ADPGK ADP-dependent glucokinase, OE overexpression

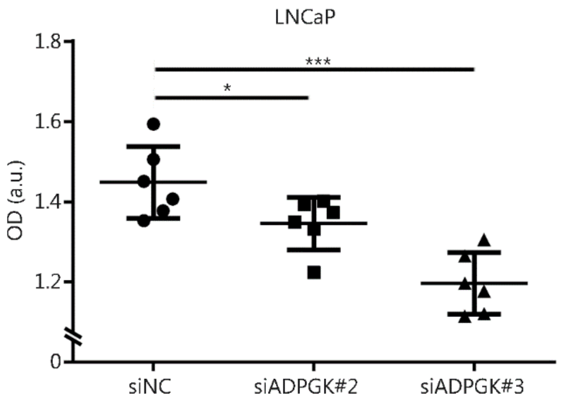

Fig. S5 The impact of *ADPGK* knockdown on LNCaP cell viability was assessed in CCK-8 assay. $^{*}P < 0.05$, $^{***}P <$ 0.001. OD optical density, NC negative control, ADPGK ADP-dependent glucokinase, a.u. artificial unit

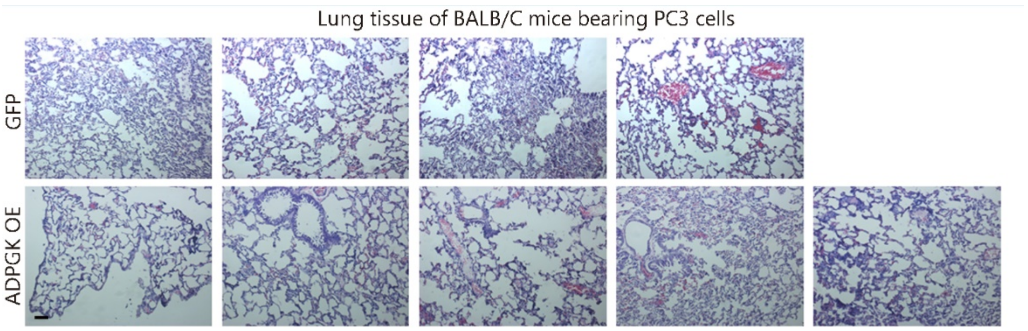

Fig. S6 Hematoxylin and eosin (HE) staining of lung tissues of mice bearing PC3 cells. Scale bar = 50 μm. ADPGK ADP-dependent glucokinase, OE overexpression
